# Diagnosis of metabolic syndrome in nursing professionals: An accuracy study

**DOI:** 10.1371/journal.pone.0295985

**Published:** 2024-06-10

**Authors:** Érica Velasco Dias Gomes, Rebeca de Souza Vasconcelos, Natália Maria Freitas Coelho, Lorena de Carvalho Almeida, Dandara Almeida Reis da Silva, Monique Magnavita Borba da Fonseca Cerqueira, Jeane Magnavita da Fonseca Cerqueira, Sarah dos Santos Conceição, Johelle de Santana Passos Soares, Lucélia Batista Neves Cunha Magalhães, Iracema Lua, Ana Claúdia Morais Godoy Figueredo, Vitória Cézar Santos Gonçalves Brito, Sandra Lúcia Fernandes, Dayanne de Aguiar Viana, Ruan Pablo Duarte Freitas, Gabriella Moreira Requião, Luiz Alberto da Silva Lima, Barbara Kraychete Hayes, Isabelle Matos Pinheiro, Maurício Mitsuo Monção, Antônio Carlos dos Santos Souza, Simone Seixas da Cruz, Antônio Marcos Tosoli Gomes, Rodrigo Fernandes Weyll Pimentel, Barbara Oliveira Nardes, Letícia Costa Lopes, Neiva Sueli Santana Gonçalves Bastos, Argemiro D’Oliveira, Magno Conceição das Mercês, Julita Maria Freitas Coelho

**Affiliations:** 1 Department of Life Sciences, University of Bahia State (UNEB), Salvador, Bahia, Brazil; 2 School of Health Sciences, University of Brasília (UNB), Brasília, Distrito Federal, Brazil; 3 School of Odontology, Federal University of Bahia (UFBA), Salvador, Bahia Brazil; 4 Department of Medicine, School of Technology and Sciences (UniFTC), Salvador, Bahia Brazil; 5 Department of Health, State University of Feira de Santana (UEFS), Feira de Santana, Bahia, Brazil; 6 School of Health Sciences, University of Brasília, Brasília, Distrito Federal, Brazil; 7 Department of Health Science, Federal University of Bahia (UFBA), Salvador, Bahia Brazil; 8 Department of Medicine, Dom Pedro II University Center (UNIDOMPEDRO), Salvador, Bahia, Brazil; 9 Department of Social Science, State University of Bahia, Serrinha, Bahia, Brazil; 10 Children’s Hospital of Los Angeles, Los Angeles, California, United States of America; 11 Department of Technology and Science, Federal Institute of Education, Science and Technology of Bahia, Salvador, Bahia, Brazil; 12 Department of Information Technology, Federal Institute of Education, Science and Technology of Bahia. Salvador, Bahia, Brazil; 13 Department of Collective Health, Federal University of Recôncavo of Bahia (UFRB), Santo Antônio de Jesus, Bahia, Brazil; 14 School of Nursing, State University of Rio de Janeiro (UERJ), Rio de Janeiro, Rio de Janeiro, Brazil; 15 University Hospital Professor Edgard Santos (HUPES), Salvador, Bahia, Brazil; 16 Institute of Health Science, Federal University of Bahia (UFBA), Salvador, Bahia Brazil; 17 Department of Teaching, Federal Institute of Education, Science and Technology of Bahia, Lauro de Freitas, Bahia, Brazil; University of Botswana School of Medicine, BOTSWANA

## Abstract

Metabolic Syndrome (MetS) represents a group of cardiovascular risk factors. This article aims to evaluate the accuracy of the tools of MetS diagnosis in Nursing professionals from Primary Health Care (PHC) in Bahia, Brazil. A cross-sectional study with a random sample selected according to essential health information for the diagnostic of MetS. For MetS diagnostic, we used EGIR, NCEP-ATPIII, AACE, IDF, Barbosa *et al*. (2006), and IDF/AHA/NHLBI (defined as gold standard) definition. Sensitivity, specificity, predictive values, and likelihood ratio were estimated for each diagnostic tool and compared with the gold standard. Kappa statistic was used to determine the agreement between the diagnostic methods. One thousand one hundred and eleven nursing professionals were included in this study. Sensitivity varied from 15% to 95.1%, and specificity varied between 99.5% and 100%. IDF and Barbosa et al. (2006) definitions were more sensitive (95.1% and 92.8%, respectively), and EGIR, NCEP, ATP III, and IDF showed 100% specificity. IDF and Barbosa *et al*. (2006) use suitable metabolic syndrome identification and confirmation criteria. The highest agreement was found in the definition of the IDF, Barbosa et al. (2006) and the NCEP ATP III. Defining metabolic syndrome with a higher diagnostic accuracy could contribute to the screening and the early identification of nursing professionals with cardiovascular disease risk factors, which provide opportunities for appropriate prevention and treatment.

## Introduction

Metabolic syndrome (MetS) is a cluster of metabolic, hemodynamic, and inflammatory disorders that include increased blood pressure, visceral obesity, insulin resistance, an increase in triglyceride levels, a decrease in HDL cholesterol levels, and dysglycemia [1,2]. It represents a worldwide epidemic and is associated with elevated morbidity and mortality rates, as it predisposes individuals to a double higher risk of death, triple higher risk of cardiovascular diseases, and a five-time higher risk of developing diabetes mellitus type 2 [3–5].

Several definitions and diagnostic criteria for MetS contribute to limited and deficient MetS prevalence information, impairing comparison between studies [6]. Aiming at overcoming these barriers, in 2009, the International Diabetes Federation (IDF) and the American Heart Association/National Heart, Lung and Blood Institute (AHA/NHLBI) proposed a new definition that considers at least three among five risk factors for MetS diagnosis. This unified tool is convenient for worldwide use in clinical practice, facilitating then the comparison of data from different countries [7].

The rising prevalence of MetS is associated with increased worldwide obesity rates and a sedentary lifestyle [7]. The global estimate for MetS prevalence ranges from 20 to 25% in the adult population [7]. In Latin America, Mets prevalence varied from 25 to 45% regarding different criteria [8]. In Brazil, a 38.4% prevalence of MetS was identified in a representative sample using the unified criteria from IDF/AHA/NHLBI [9].

Research has shown that MetS is associated with work activity and is influenced by work in shifts, job conditions, type of occupation, stress, and burnout [10–19]. The relation between MetS and work conditions has been considered by the fact that working in shifts, including the night shift, physical and psychological overload related to the work activities might contribute to changes in circadian and hormonal rhythms in the sympathetic nervous system and neuroendocrine system. These changes predispose individuals to high blood sugar, an increase in abdominal fat, an increase in blood pressure, sedentarism, as well as other metabolic changes [19–22].

In the face of above mentioned, nursing professionals stand out since work conditions for this category have been favoring the development of MetS. Such development comes whether directly from changes in the physiological system due to nightshifts, work overload and less control of them, or indirectly in virtue of unhealthy behaviors [16–19].

This is increasingly relevant because nursing professionals represent 59% of the labor force in the health field worldwide [23]. Moreover, among high-education professionals, nurses are the second one in job post occupancy [24].

Considering that the nursing professionals need to have a good health quality to ensure safety and quality in patient care and that there are few studies involving this occupational group, new studies are needed to diagnose MetS and contribute to the provision of preventive measures in this population. In addition, investigations for identifying adequate criteria to screen or confirm the diagnosis of MetS in this group are necessary, with the possibility of expanding the results to population in general. It should be emphasized that, to date, no study on the diagnostic accuracy of MetS involving six different criteria has been identified in the literature, even in the professional category investigated.

Therefore, this study aimed to evaluate the diagnostic accuracy of the diagnostic tools for metabolic syndrome among nursing professionals from Primary Health Care (PHC) in Bahia, Brazil.

## Materials and methods

### Study design

The study is an analytical and validation cross-sectional analysis. The data comes from a population-based epidemiologic and multicentric survey performed at health units from PHC in the state of Bahia, Brazil, between March 07, 2017 to January 23, 2018 [15,25,26]. Data collection was authorized by the Research Ethics Committee from the State University of Bahia, Brazil, through the report n° 872.365/ 2014. The Declaration of Helsinki of the World Medical Association and Legal Resolution 466/2012 of Brazil were fully respected. We obtained written consent from all participants by using a free informed consent form whose copies are archived at the State University of Bahia. To guarantee the confidentiality and anonymity of the participants, a number was assigned to each individual.

We adopted the recommendations from the Standards for Reporting Diagnostic Accuracy- STARD [27].

### Participants

The study population is consisted of nursing professionals from PHC in Bahia, Brazil. Bahia is the fourth most populous Brazilian state, consisting of 417 municipalities divided into seven mesoregions. A random selection of 10% of the municipalities in each mesoregion was carried out by using Microsoft Office Excel 2010, totaling 43 municipalities [25]. All PHC nursing professionals from the 43 selected municipalities (1,195 individuals) were invited to participate in the study.

Eligibility criteria were defined based on components needed for the MetS diagnosis according to the six criteria used and include the following: information on previous treatment/diagnosis of systemic arterial hypertension and diabetes mellitus; presence/absence of cardiovascular problems, polycystic ovary syndrome (for women), non-alcoholic fatty liver disease or *Acanthosis nigricans*; sedentarism, weight and height information, waist circumference, body mass index, and blood pressure; fasting glucose, triglycerides, and high-density cholesterol laboratory results.

The following individuals were excluded from this study: individuals on sick leave; with less than 6 months of experience in PHC; in solely administrative work routines; pregnant women; women in their menstrual period; and individuals with a diagnosis of depression, anxiety, and Burnout Syndrome before occupying the current position, and liver cirrhosis, alcohol and drug addiction [15,25,26]. Among the total of 1,195 nursing professionals invited, 22 refused to participate in the study and 48 were considered ineligible due to some exclusion criteria. Within the 1,125 potentially eligible participants, there were 14 losses due to lack of information in the database, a value lower than the 20% considered in the sample calculation and acceptable for this type of research. The final sample was consisted of 1,111 participants.

No robust studies were found in the literature that have explored the MetS rates among PHC nursing professionals, which led us to conduct a pilot study in a similar population to obtain parameters for sample calculation. The MetS rates are 20% and 33.3% in the non-exposed and exposed groups, respectively. We performed a sample size estimation for this investigation to ensure representativeness from the available sample. We used an online calculator from the University of Sao Paulo (USP) available on http://estatistica.bauru.usp.br/calculoamostral/calculos.php and, based on this calculation, we reached a minimum and representative sample of 624 individuals. We used the following parameters: expected sensitivity and specificity of 90%, 95% confidence level, 5% error, and MetS prevalence of 33.3% in the population according to results from a pilot study at baseline investigation [25]. We then increased the sample by 20% (n = 125) to compensate possible losses and by 50% (n = 312) to correct the design effect. Therefore, 1,061 PHC nursing professionals were obtained.

### Proceedings and variable evaluation

Anamnesis and data collection were performed through individual interviews in a reserved office inside each health care units, between 2017 and 2018, by using a record sheet previously tested in order to obtain information on socioeconomic, demographic, occupational, and lifestyle information, as well as biological aspects of human life information, such as: the presence or absence of polycystic ovary, *Acanthosis nigricans*, non-alcoholic fatty liver disease, diabetes, arterial hypertension, and cardiovascular problems. Moreover, weight, height, waist circumference, blood pressure were measured, and blood samples for clinical laboratory examinations were collected.

To guarantee homogeneity in the application of the record sheets, a calibration was carried out among the research assistants, by interviewing thirty hospital professionals. The concordance among the assistants was calculated by using the Kappa index. A value of 0.87 was found and considered acceptable.

For weight measurement, the professional was asked to wear as few garments as possible and to be barefooted. A Welmy^®^ digital anthropometric scale with a maximum capacity of 200 kilograms-kg was used. Height was measured with a retractable aluminum stadiometer, measuring up to 2 meters-m with a 0.5 cm graduation. The individual was asked to remain still, standing with the back against the device, the spine erect and the head in the Frankfurt plane [28].

For waist circumference, we obtained the mean value between two measurements at the midpoint of a horizontal distance between the lower edge of the costal arch and the iliac bone. The participant remains in the orthostatic position, with arms along the body, feet together, and weight divided between legs and face in a straight position, using an inelastic, glazed measuring tape divided into 0.1 centimeters, from ISP ^®^ (Wiso, Santa Tereza, Paraná, Brazil) [25].

Arterial blood pressure was measured by a trained professional using a stethoscope (Littmann ^®^, Classic III, 3M, USA) and an aneroid sphygmomanometer (BD ^®^ adult medium size, USA) previously calibrated. The participants were instructed to rest at least five minutes beforehand in a calm environment under the following instructions: not having a full bladder; not having practiced physical exercise 60 to 90 minutes before; not consuming alcoholic beverages, coffee, or food; not having smoked 30 minutes before; keeping their legs uncrossed, feet flat on the floor, back leaning back in the chair, relax and not talk during the measurement. After five minutes of rest, two measurements were made on the nursing professional′s left upper limb. The first Korotkoff sound was considered to read systolic blood pressure and the last to read diastolic blood pressure. The mean value was obtained between two measurements in five minutes [25].

Blood samples were collected after 12-hour fasting and analyzed by a reference laboratory in each municipality. Serum fasting glucose, HDL-cholesterol, and triglyceride levels were measured using standard enzymatic and colorimetric techniques.

### Diagnostic tools of metabolic syndrome

Participants were subdivided regarding presence or absence of MetS according to six different diagnostic criteria, in which five are proposed by international organizations: European Group of Resistance Insulin (EGIR) [29], Adult Treatment Panel III from National Cholesterol Education Program (NCEP-ATPIII) [30], American Association of Clinical Endocrinologists (AACE) [31], International Diabetes Federation (IDF) [3] and International Diabetes Federation/ American Heart Association/ National Heart, Lung and Blood Institute (IDF/AHA/NHLBI) [7] and one national criterion proposed by Barbosa *et al*. in 2006 [32], which considers criteria adopted by NCEP-ATPIII, although they adopt a different central obesity cut-off value, specific for Brazilian population, which became an adapted IDF criteria.

**Fig 1:**
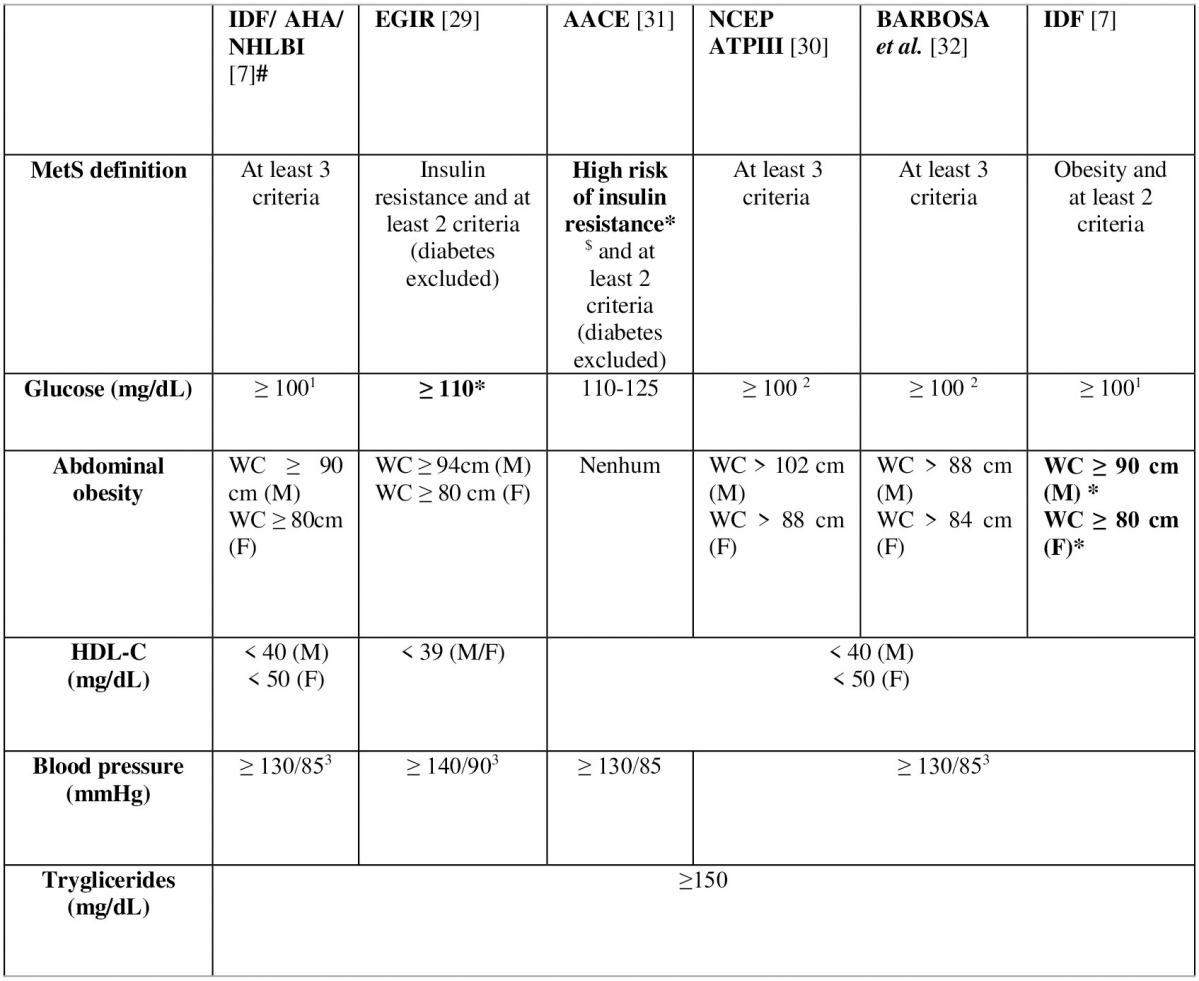
MetS diagnostic criteria in adults, from IDF/AHA/NHLBI, EGIR, AACE, NCEP ATPIII, Barbosa et al. (2006) [32] and IDF. # Reference criteria–the gold standard- *Mandatory criteria for diagnostics. ^$^High risk of insulin resistance is indicated by the presence of at least one of the factors: Cardiovascular disease diagnosis, hypertension, polycystic ovary syndrome, non-alcoholic fatty liver disease or *Acanthosis nigricans*; family history of type 2 diabetes, hypertension or CVD; history of gestational diabetes or glucose intolerance; non-white ethnicity; sedentary lifestyle; being older than 40 years old; BMI ≥ 25 kg/m^2^ or WC >94 cm for men e > 80 cm for women ^1^ “or previous DM diagnosis.”^2^ “or treatment for DM.”. ^3^ “or treatment for SAH.” M: Male. F: Female. EGIR: European Group for the Study of Insulin Resistance; AACE: American Association of Clinical Endocrinologists; NCEP ATP III: National Cholesterol Education Program Adult Treatment Panel III; IDF: International Diabetes Federation; AHA: American Heart Association; NHLBI: National Heart, Lung e Blood Institute; CVD: Cardiovascular Disease; DM: Diabetes *Mellitus*; HDLc: High-Density Lipoprotein cholesterol; BMI: Body Mass Index; WC: Waist Circumference; SAH: Systemic Arterial Hypertension.

Each definition has its criteria and is analyzed by five components: abdominal obesity, high-density lipoprotein cholesterol (HDL), triglycerides, glycemia, and blood pressure. These criteria have variable cut-offs, and their respective parameters are presented in Fig 1.

IDF/AHA/NHLBI criteria, also known as the harmonized criteria, was selected as gold standard since it was built to unify different existing criteria. Thus, it aimed at adopting one definition to facilitate its use in clinical practice, with cutoff values according to different ethnic and gender groups, with the potential for comparison among international studies.

### Statistical analysis

We conducted an univariate analysis to characterize the sample according to socioeconomic, demographic, lifestyle aspects and clinical characteristics stratified by sex, and estimated the outcome frequency by the six criteria selected.

The descriptive analysis was stratified by sex, given that there are some cutoff points with different values for men and women. Absolute and relative frequencies were obtained for categorical variables and mean and standard deviations for continuous variables. Initially, the Kolmogorov-Smirnov test was performed to evaluate the distribution of data for continuous variables, and based on the presence or absence of normality, it was defined as to the use of the mean or median and the use of the T test or the Mann-Whitney test for continuous variables analysis. Pearson chi-square test was used for bivariate analysis among sex categories, and p-value ≤0.05 as a reference value. The T-test or Mann-Whitney test was used for continuous variables analysis, according to data normality measured by the Kolmogorov-Smirnov test.

We emphasize that the differences by sex were not significant and, therefore, do not justify stratified accuracy analyses.

Lastly, we compared the six MetS diagnostic criteria with the (IDF/AHA/NHLBI) gold standard. We calculated diagnostic values and their 95% confidence intervals: sensitivity (Se), specificity (Sp), positive predictive value (PPV), negative predictive value (NPV), positive likelihood ratio (LR+), and negative likelihood ratio (LR-). Where, Se = a/ a+c, Sp = d/ b+d, PPV = a/ a+b, NVP = d/ c+d, LR+ = Se/(1-Sp) and LR- = (1-Se)/Sp, where a = true-positive, b = false-positive, c = false-negative, and d = true-negative [33].

Kappa agreement test was conducted to evaluate the answers provided in the tests. Values lower than 20% were considerate slight agreement, 21% to 40% were considered to fair agreement, 41% to 60% were classified as moderate agreement, 61% to 80% were considered to have substantial agreement and above 80% to have almost perfect agreement.

Data analysis was executed using STATA (Data Analysis and Statistical Software), versions 11.2 and 18.

## Results

**Fig 2:**
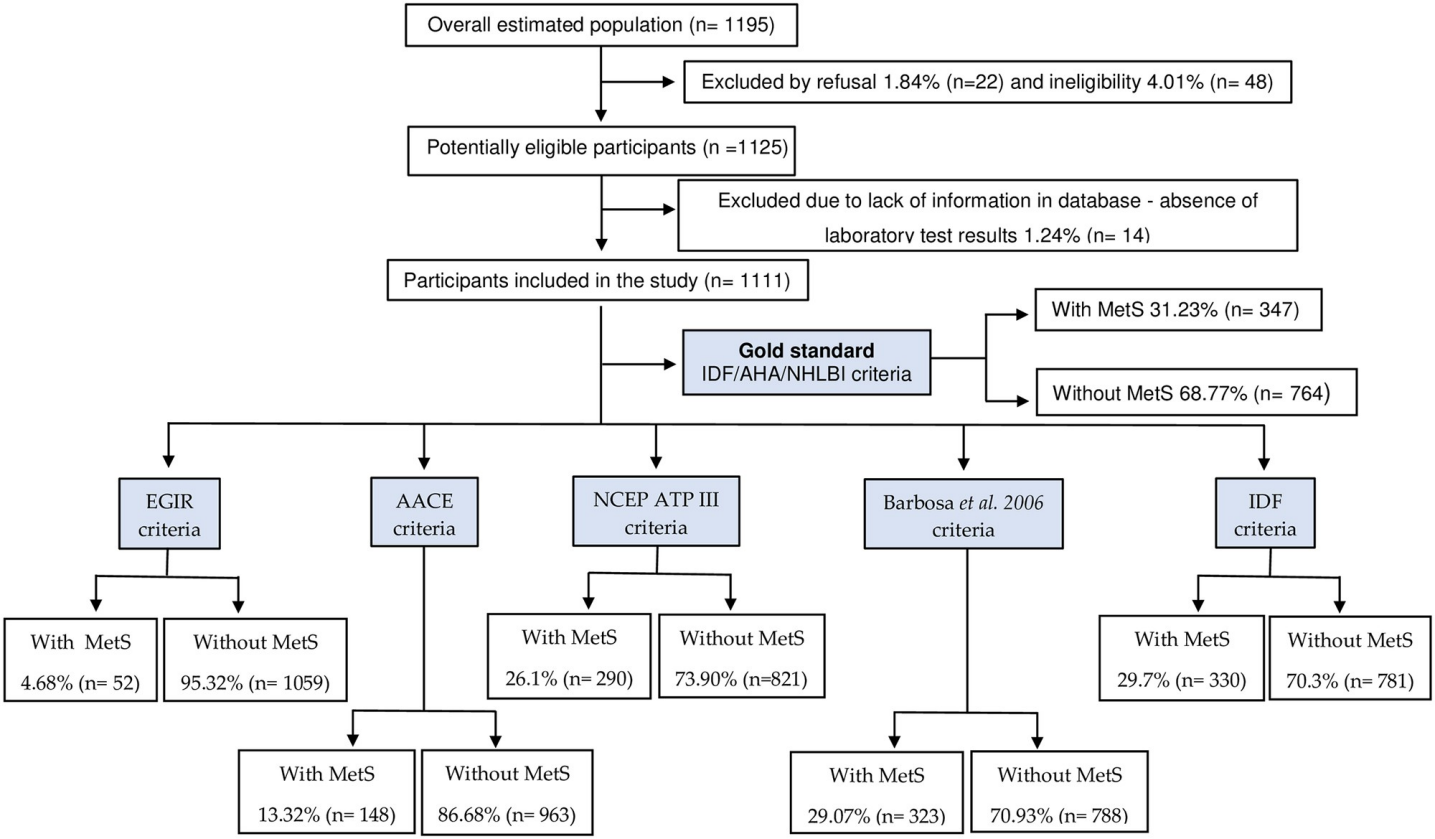
Flow diagram of study design and metabolic syndrome classification according to six different diagnostic tools.

A flowchart showing the inclusion of participants in each phase is presented (Fig 2).

The final analyzed sample comprised 1,111 nursing professionals from PHC, who are mostly young (up to 35 years old) (52.2%), Black people (74.8%), female (87,7%), residents in urban areas (83.62%), with a partner (53.83%) and with offspring (59.5%), nursing technicians (59.32%), with income higher than three minimum wages (53.92%), non-smokers (88.03%), that consumed alcoholic beverages (63.37%) and performed physical activity (56.71%) (Table 1).

**Table 1:** Socioeconomic-demographic, lifestyle and clinical characteristics according to sex in nursing professionals from Primary Health Care in Bahia (n = 1111). Bahia, Brazil.

Characteristics	Total	Sex		
	n (%)	Female n (%)	Male n (%)	p-value*
Age (years old)				
Up to 35 years	579 (52.12)	504 (51.69)	75 (55.15)	0.450
36 years old or mores	532 (47.88)	471 (48.31)	61 (44.85)	
Race				
Non black people	280 (25.20)	242 (24.82)	38 (27.94)	0.432
Black people	831 (74.80)	733 (75.18)	98 (72.06)	
Place of residence				
Rural area	182 (16.38)	143 (14.67)	39 (28.68)	<0.01*
Urban area	929 (83.62)	832 (85.33)	97 (71.32)	
Marital status				
With a partner	598 (53.83)	528 (54.15)	70 (51.47)	0.557
Without a partner	513 (46.17)	477(45.85)	66 (48.53)	
Offspring				
With offspring	661 (59.50)	592 (60.72)	69 (50.74)	0.189
Without offspring	450 (40.50)	383 (39.28)	67 (49.26)	
Professional category				
Nurse	452 (40.68)	388 (39.79)	64 (47.06)	0.106
Nursing technician	659 (59.32)	587 (60.21)	72 (52.94)	
Family income^1^				
Up to two minimum wages	512 (46.08)	459 (47.08)	53 (38.97)	0.076
Three or more minimum wages	599 (53.92)	516 (52.92)	83 (61.03)	
Smoking habit				
No	978 (88.03)	883 (90.56)	95 (69.85)	<0.01*
Yes	133 (11.97)	92 (9.44)	41 (30.15)	
Consumes alcohol beverages				
No	407 (36.63)	374 (38.36)	33 (24.26)	0.001*
Yes	704 (63.37)	601 (61.64)	103 (75.74)	
Practice of physical activities				
Yes	630 (56.71)	545 (55.90)	85 (62.50)	0.145
No	481 (43.29)	430 (44.10)	51 (37.50)	
Fasting glucose^2^^,^^3^^,^^4^^,^^5^				
Positive (≥ 100)	186 (16.74)	159 (16,31)	27 (19.85)	0.300
Negative (<100)	925 (83.26)	816 (83.69)	109 (80.15)	
Fasting glucose^6^				
Positive (≥ 110)	83 (7.47)	67 (6.87)	16 (11.76)	0.042*
Negative (<110)	1028 (92.53)	908 (93.13)	120 (88.24)	
Waist circumference ^2^^,^^5^				
Positive (≥ 90 (M) ≥ 80 (F))	737 (66.34)	666 (68.31)	71 (52.21)	<0,01*
Negative (< 90 (M) < 80 (F))	374 (33.66)	309 (31.69)	65 (47.79)	
Waist circumference^6^				
Positive (≥ 94 (M) ≥ 80 (F))	721 (64.90)	666 (64.90)	55 (40.44)	<0,01*
Negative (< 94 (M) < 80 (F))	390 (35.10)	309 (31.69)	81 (59.56)	
Waist circumference^3^				
Positive (>102 (M) > 88 (F))	459 (41.31)	419 (42.97)	40 (29.41)	0.003*
Negative (≤ 102 (M) ≤ 88 (F))	652 (58.69)	556 (57.03)	96 (70.59)	
Waist circumference^4^				
Positive (> 88 (M) > 84 (F))	596 (53.65)	523 (53.64)	73 (53.68)	0.994
Negative (≤ 88 (M) ≤ 84 (F))	515 (46.35)	452 (46.36)	63 (46.32)	
HDLc^2^^,^^3^^,^^4^^,^^5^^,^^7^				
Positive (< 40 (M) < 50 (F))	489 (44.01)	458 (46.97)	31 (22.79)	<0,01*
Negative (≥40 (M) and ≥ 50 (F))	622 (55.99)	517 (53.03)	105 (77.21)	
HDLc^6^				
Positive (< 39)	208 (18.72)	184 (18.87)	24 (17.65)	0.732
Negative (≥ 39)	903 (81.28)	791 (81.13)	112 (82.35)	
Blood pressure^2^^,^^3^^,^^4^^,^^5^^,^^7^				
Positive (≥ 130/85)	197 (17.73)	160 (16.41)	37 (27.21)	0.002*
Negative (< 130/85)	914 (82.27)	815 (83.59)	99 (72.79)	
Blood pressure^6^				
Positive (≥ 140/90)	121 (10.89)	94 (9.64)	27 (19.85)	0.002*
Negative (< 140/90)	990 (89.11)	880 (90.36)	109 (80.15)	
Triglycerides^2^^,^^3^^,^^4^^,^^5^^,^^6^^,^^7^				
Positive (≥ 150)	371 (33.39)	315 (32.31)	56 (41.18)	0.040*
Negative (< 150)	740 (66.61)	660 (67.69)	82 (58.82)	

Source: Authors.

^1^ Sum of income of all family members. Minimum wage in 2017 was BR R$ 937.00 or US$ 286.54 at the time. In 2018 the minimum wage was BR R$ 954.00 or US$ 261,36.

^2^Cutoff point IDF/AHA/NHLBI.

^3^Cutoff point NCEP ATPIII.

^4^Cutoff point Barbosa et al. (2006) [32].

^5^Cutoff point IDF.

^6^Cutoff point EGIR.

^7^Cutoff point AACE. M: Male, F: Female. HDLc- High Density Lipoprotein Cholesterol

* statistically significant p <0,05.

Considering the clinical characteristics of these nursing professionals from PHC and the parameters used to evaluate MetS through different diagnostic tools, it is verified that most participants do not present changes in fasting blood glucose, blood pressure, high triglycerides or low HDLc. However, with regard to waist circumference, most participants have values higher than the reference values for the criteria used, except for the values used by NCEP ATP III, where the majority of participants (58.69%) do not present changes in waist circumference (Table 1).

Overall, both sexes showed homogeneity regarding studied variables. Regarding socioeconomic-demographic and lifestyle characteristics, there were statistically significant differences between sexes for the following variables: the place of residence, smoking habits, and consumption of alcoholic beverages. Regarding the clinical characteristics of the participants, statistically significant differences were also found for fasting blood glucose values ≥ 110 mg/dL, waist circumference values greater than 90 cm for male and 80 cm for female, HDLc levels < 40 mg/dL for male and < 50 mg/dL for female, triglycerides levels ≥ 150mg/dL, blood pressure levels ≥ 130x85 or ≥ 140/90 (Table 1).

**Fig 3:**
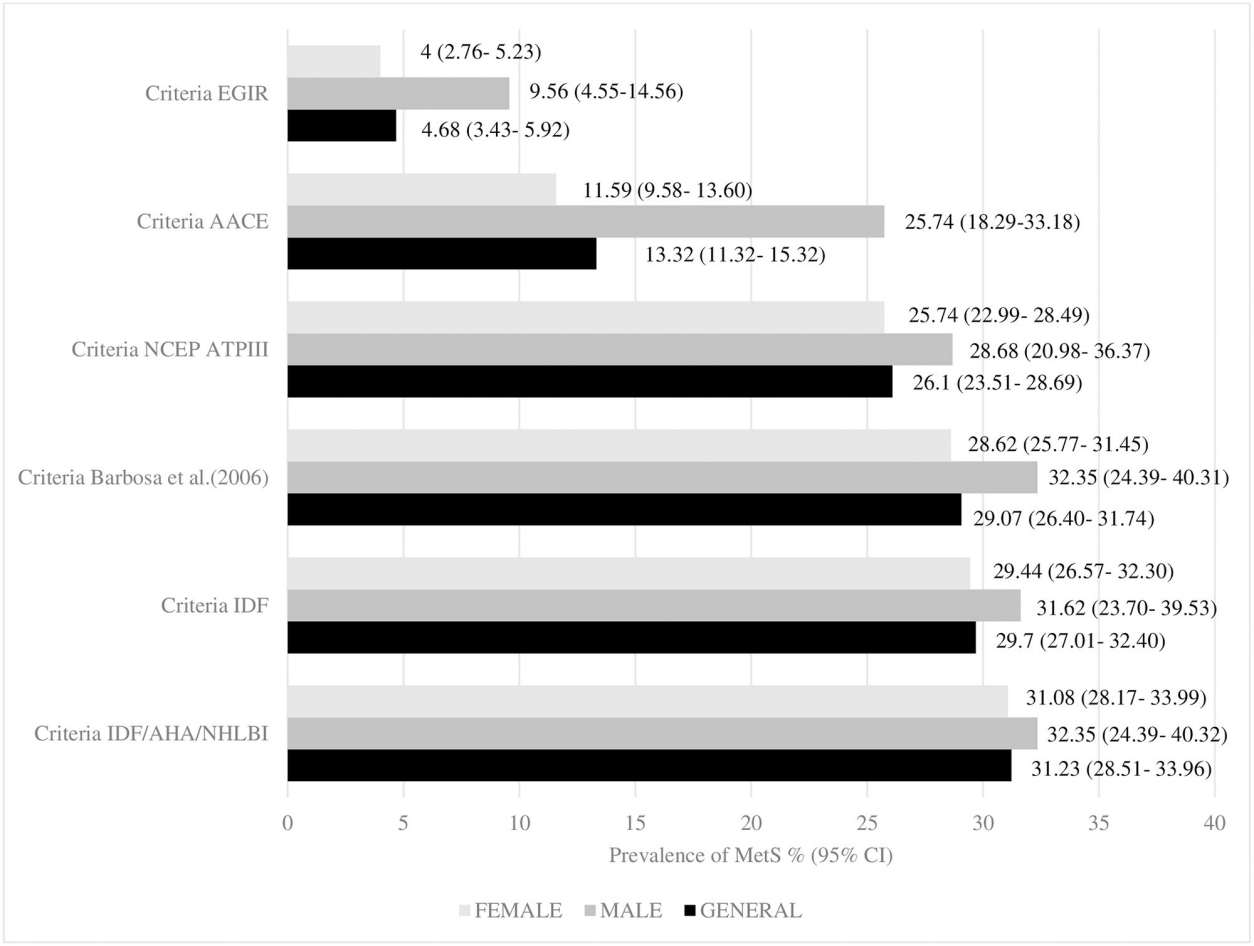
The overall and by sex prevalence of MetS in nursing professionals from Primary Health Care in Bahia and its confidence intervals, according to six different diagnostic tools (n = 1111). Bahia, Brazil.

The overall prevalence of MetS among nursing professionals from PHC in Bahia varied between 4.68% and 31.23%, showing differences according to the criteria used. Regarding the gold standard criteria, the prevalence of MetS was 31.23% (n = 347), whereas comparison criteria showed different values: IDF 29.70% (n = 330), NCEP ATP III 26.10% (n = 290), Barbosa *et al*. (2006) [32] 29.07% (n = 323), EGIR 4.68% (n = 52) and AACE 13.32% (n = 148) (Fig 3).

Considering the sex, the prevalence of MetS in male nursing professionals varied between 9.56% (n = 13) according to the EGIR Criteria and 32.35% (n = 44) according to the Barbosa et al. criteria (2006) [32] and IDF/AHA/NHLBI. Among female nursing professionals, the prevalence of MetS varied between 4.0% (n = 39) according to the EGIR criteria and 31.08% (n = 303) according to the IDF/AHA/NHLBI Criteria (Fig 3).

Regarding clinical characteristics, male nursing professionals showed higher glycemia and triglyceride rates, waist circumference, BMI, hypertension, and lower HDL cholesterol rates than female nursing professionals. Most clinical characteristics showed statistically significant differences, except for glycemia and HDLc levels (Table 2).

**Table 2:** Clinical characteristics from nursing professionals from Primary Health Care in the Bahia (n = 1111). Bahia, Brazil.

Clinical characteristics	Sex				p-value*
	Female (n = 975)		Male (n = 136)		
	Mean	SD	Mean	SD	
Fasting glucose (mg/dL)	84.48	18.04	86.19	16.08	0.09
Triglycerides (mg/dL)	132.41	55.24	148.17	67.27	<0.01*
HDLc (mg/dL)	58.80	31.30	57.72	27.02	0.63
Waist circumference (cm)	86.33	14.11	89.60	15.71	<0.01*
BMI (Kg/m^2^)	26.18	8.64	27.55	4.50	<0.01*
Systolic blood pressure (mmHg)	116.58	15.44	123.70	14.09	<0.01*
Diastolic blood pressure (mmHg)	77.02	10.61	82.55	9.41	<0.01*

Source: Authors.

SD = Standard deviation; HDLc- High Density Lipoprotein cholesterol; BMI—Body Mass Index;

*statistically significant (p<0.05).

**Fig 4:**
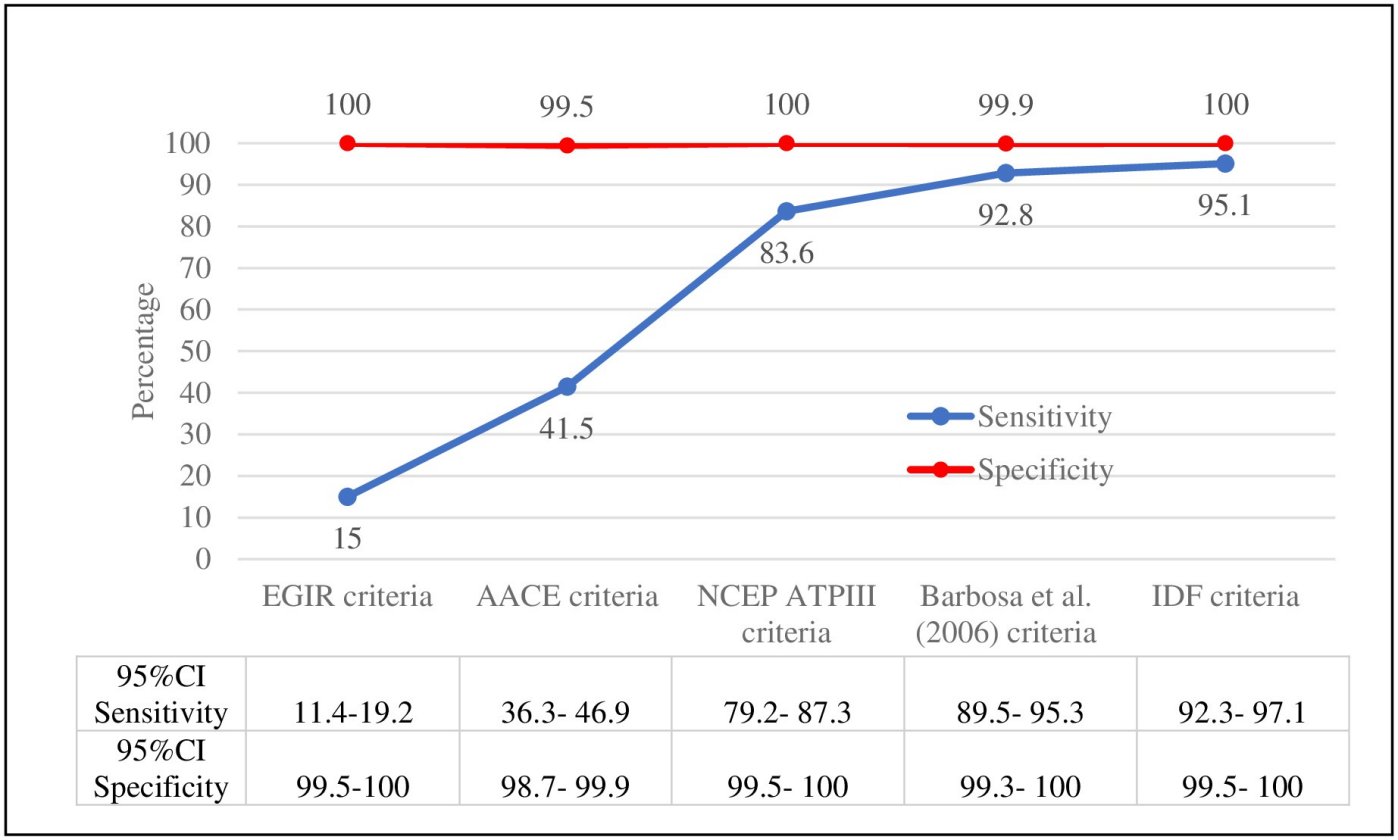
Values of sensitivity and specificity and their confidence intervals, according to IDF/AHA/NHLBI criteria for the diagnostic of MetS (n = 1111). Bahia, Brazil.

IDF/AHA/NHLBI criteria was considered the gold standard, the sensitivity from the comparison criteria varied from 15% according to the EGIR Criteria to 95.1% according to the IDF Criteria, while specificity varied from 99.5% considering to the AACE Criteria to 100% considering to the EGIR Criteria, NCEP ATP III Criteria and IDF Criteria (Fig 4).

Regarding positive predictive values, they varied from 97.3% (AACE Criteria) to 100% (EGIR, NCEP ATPIII and IDF Criteria), showing the percentage of participants positive to MetS that had it. Negative predictive values varied from 72.1% (EGIR Criteria) to 97.8% (IDF Criteria), showing the proportion of participants who were negative for MetS and did not have it (Table 3). It is worth noting that the predictive values were calculated based on the prevalence of the IDF/AHA/NHLBI gold standard.

**Table 3:** Validity indicators, with confidence intervals, from EGIR, AACE, NCEP ATP III, BARBOSA *et al*. (2006) [32], and IDF criteria, with IDF/AHA/NHLBI as a gold standard criteria for the diagnosis of MetS in nursing professionals in Bahia, Brazil.

	Index tests % (95% CI)				
Definition	EGIR	AACE	NCEP ATP III	Barbosa *et al*. (2006) [32]	IDF
Positive predictive value	100 (93.2–100)	97.3 (93.2–99.3)	100 (98.7–100)	99.7 (98.3–100)	100 (98.9–100)
Negative predictive value	72.1 (69.3–74.8)	78.9 (76.2–81.5)	93.1 (91.1–94.7)	96.8 (95.4–97.9)	97.8 (96.5–98.7)
Positive likelihood ratio	-	79.3 (29.6–212)	-	709 (100–5028)	-
Negative likelihood ratio	0.85 (0.81–0.89)	0.59 (0.54–0.64)	0.16 (0.13–0.21)	0.07 (0.04–0.10)	0.05 (0.31–0.78)

Source: Authors.

95% Confidence Interval.

- Estimate not obtained due to the absence of false positives.

We highlight that it was impossible to estimate the positive likelihood ratio for EGIR, NCEP ATP III, and IDF criteria from the defined gold standard since we did not identify false positive results. Regarding other criteria, they showed positive likelihood ratios higher than one, which supports the presence of the disease, hence, indicates a higher chance of a positive result among those with the disease when compared to those without, emphasizing the criteria of Barbosa et al. (2006) [32] as the one with the best performance (Table 3).

As for the negative likelihood ratio, most criteria had a good performance with values close to zero, with a slight chance of presenting a negative result among individuals with MetS compared to those without, with the IDF criteria standing out (Table 3).

Most of the criteria (IDF, Barbosa et al (2006) [32] and NCEP ATPIII) present an almost perfect or substantial agreement with the IDF/AHA/NHLBI criteria, except for the AACE criteria, which have a fair level of agreement and the EGIR criteria, which have slight agreement (Table 4).

**Table 4:** Agreement of MetS diagnostic criteria with the IDF/AHA/NHLBI criteria (gold standard), Bahia, Brazil.

Criteria	Kappa (k)	Agreement
EGIR	0.1951	Slight
AACE	0.4858	Moderate
NCEP ATPIII	0.8750	Substantial
Barbosa et al. (2006) [32]	0.9445	Almost perfect
IDF	0.9639	Almost perfect

## Discussion

### Main findings

To the best of our knowledge, this is the first study to compare five different diagnostic criteria for MetS in nursing professionals, based on the IDF/AHA/NHLBI gold standard. Our findings indicate that the accuracy of the IDF criteria for diagnostic of MetS was the highest among the criteria evaluated. While evaluating different criteria, they all showed high specificity taking the gold standard as a reference. Variation went from 99.5% to 100%, indicating all or almost all individuals without MetS do not fit the defined criteria for the diagnostic of MetS. Thus, they do have a negative test.

These findings are supported by the fact that sensitivity and specificity are the two most used diagnostic precision measures, and they show how good the performance of a diagnostic test is compared to a gold standard [34,35].

More specific criteria show a better MetS confirmation strength and, on average, a lower sensitivity. IDF, Barbosa, et al. (2006) [32], and NCEP ATP III criteria are more sensitive, which is the reason why they are the most indicated for diagnostic screening of the syndrome. Regarding specificity, all criteria were similar, which makes them an excellent diagnostic confirmation method for MetS.

In summary, the results from this study show that IDF, Barbosa et al. (2006) [32], and NCEP ATP III were the best criteria for identifying and confirming the syndrome. They simultaneously showed good sensitivity and specificity and, thus, a reasonable probability of detecting MetS. In addition to presenting excellent kappa agreement.

### Secondary findings

A relatively high MetS prevalence was detected in the studied group, independently of the criteria used, with the exception only of the EGIR criteria. Similarly, from studies performed worldwide, the prevalence of MetS showed a high variation depending on the adopted criteria for the analysis [8,36–38]. It may be justified by different cutoff values for components and combinations between them according to different MetS definitions, or even the absence of the waist circumference parameter as per AACE criterion.

Regarding the overall MetS prevalence in this study, we observed that for most criteria (NCEP ATP III, IDF, Barbosa *et al*., and IDF/AHA/NHLBI), the MetS prevalence was higher than the one estimated in 2006 for the adult world population, i.e., 20% to 25% [3]. It is worth emphasizing that the prevalence of MetS according to the harmonized criteria (31.23%), considered as the gold standard in this study, was close to the estimate (38.4%) of MetS found in the first national study using this same criteria, laboratory data and a representative sample of the Brazilian population [9].

Analyzing the clinical characteristics of men and women, we found that among the five components considered for the diagnosis of MetS, changes in blood pressure and triglyceride values with statistical significance were more common in men, while among women the changes in HDLc values were more prominent, except for cutoff point and waist circumference in the EGIR criteria, and the cutoff point in the Barbosa et al. criteria. In face of this findings, it was verified that women had better metabolic parameters than men. This result was different in relation to another study with a representative sample of the Brazilian population that showed opposite results, where men had better metabolic parameters than women [9]. It is worth emphasizing that women also showed better lifestyle habits, except for practice of physical activity. These results show the need to intensify healthcare strategies for the male nurses. Other secondary findings are related to other precision diagnostic measures, for instance, positive and negative predictive values and likelihood ratios, due to the importance of judgment of post-test probability.

Overall, the predictive values found that most criteria tested showed promising results. Negative predictive values of EGIR and AACE criteria showed high false positives for these tests. That is probably because this condition adds a high positive predictive value. Regarding the positive likelihood ratio, the criterion from Barbosa et al. showed a better performance, while IDF had a better performance for the negative likelihood ratio.

### Strengths

This study is the first in Brazil to analyze the diagnostic accuracy of the six criteria of MetS and its prevalence in one representative sample of nursing professionals. Another differential was the inclusion of EGIR and AACE criteria, which, due to their specificities and need for clinical evaluation, are only sometimes adopted in either clinical practice or research.

Therefore, our data might support the priority use of IDF or Barbosa et al. criteria for studies involving nursing professionals from PHC when the harmonized criteria is not possible, and probably, for other population subgroups.

### Study limitations

Among limitations, we can highlight bias related to health workers and incomplete data, for instance, the absence of laboratory tests for oral glucose tolerance. Moreover, the participants were not questioned regarding treatment for dyslipidemia, family history of type 2 diabetes, hypertension or cardiovascular disease, history of gestational diabetes, or glucose intolerance. These aspects may have underestimated the prevalence of MetS in some criteria.

The lack of studies on the diagnostic accuracy of MetS, specifically in nursing professionals, also represented another important limitation, as it prevented comparison with data from other locations and even evaluation of the best criteria to be used as the gold standard in this occupational group.

### Implications for clinical practice

The choice of a diagnostic test for MetS may be favorable to a more assertive screening and early identification of individuals from the overall population with risk factors for cardiovascular diseases, i.e., arterial hypertension, diabetes mellitus, obesity, and others. Thus, the findings shown in this study could be extrapolated for other population subgroups out of nursing professionals from PHC.

We expect to strengthen the primary prevention of cardiometabolic diseases, especially with an incentive for change in lifestyle, promoting physical activity, adoption of healthy eating habits, improvement of work conditions, and development of healthcare programs for the PHC professionals. Increased interest in health monitoring and professional safety with diagnostic procedures and medical referrals is essential to reduce complications and hospital admissions caused by cardiovascular diseases and absenteeism.

Moreover, the accuracy results obtained may be used as parameters for new population-based studies in Brazil regarding electing the best diagnostic test for MetS considering the specific anthropometric profile. Validating cutoff values of specific waist circumference for the Brazilian population is essential to improve the0 precision of diagnosis.

Among proposed future studies, we highlight a follow-up of the population evaluated in this study to monitor outcomes, i.e., hospital admission and general mortality or cardiovascular problems, in order to identify which criteria can predict morbimortality with higher strength.

## Conclusions

We conclude in this study that the IDF criteria, Barbosa et al. (2006) [32] criteria, and NCEP ATP III Criteria were the best tools for identifying and confirming the metabolic syndrome in nursing professionals from PHC in Bahia, Brazil. It is worth noting that they simultaneously showed good sensitivity and specificity, therefore, a good probability of detecting MetS.

The MetS prevalence in nursing professionals from PHC in Bahia, Brazil, can be considered of high magnitude regarding different consequences inherent to this disease. Regarding diagnostic accuracy by using the IDF/AHA/NHLBI criteria as a gold standard, relevant variations show the need for efforts to harmonize the currently adopted criteria.

Lastly, in the face of the results obtained, we recommend that, in the impossibility of using the harmonized criteria considered as a gold standard, IDF and Barbosa et al. criteria should be prioritized for screening and diagnosing MetS in nursing professionals from Bahia. These two criteria may result in higher MetS detection rates in the population studied, so contributing to prevent complications and provide timely treatment and interventions.

## Supporting information

S1 File Dataset metabolic syndrome in nursing professionals. (XLSX)

S2 File Code book metabolic syndrome in nursing professionals. (PDF)

S3 File Statistical analysis. (PDF)
