# Supplementary material for: Diagnosis of metabolic syndrome in nursing professionals: An accuracy study
Source: PLoS One. 2024 Jun 10;19(6):e0295985. doi: 10.1371/journal.pone.0295985 (PMC11164393; doi:10.1371/journal.pone.0295985)
Supplement: S2 File — (PDF) [file pone.0295985.s002.pdf]

PROJECT: "*BURNOUT* SYNDROME AND METABOLIC SYNDROME IN PRIMARY HEALTH CARE NURSING PROFESSIONALS"
CODE BOOK
Sociodemographic, Labor, Lifestyle and Human Biology Questionnaire

**Questionnaire number (NQUEST)**
Ascending order – cardinal number
**Profession (PROF)**
0 Nurses 1 Nursing Technician / Auxiliary
**Age in years (IDAD)**
0 Up to 35 years 1 36 years or more
**Sex (SEX)**
0 Male 1 Female
**Residence (RESID2)**
0 Rural area 1 Urban area
**Self-reported race/ (COR2)**
0 Non-black or undeclared 1 Black
**Level of education (ESCOL)**
01 Technical Level 02 Bachelor's Degree 03 Specialization 04 Master's Degree 05 Doctorate
**Family income in minimum wages (RENDA2)**
0 3 or more minimum wages 1 Up to 2 minimun wages
**Marital Status (ESTCI2)**

0 with a partner 1 without a partner
**Number of children (NFIL2)**
0 without children 1 with children
**Number of dichotomized children (NFILDIC)**
0 to 2 children 1 3 or more children
**You have hypertension (HIPERTDIC)**
0 No 1 Yes
**You have Diabetes (DIABDIC)**
0 No 1 Yes
**You have cardiovascular problems (CARDDIC)**
0 No 1 Yes
**You have polycystic ovary (OVPOLI)**
0 No 1 Yes 04 Not applicable
**Have you ever felt the need to undergo psychiatric or psychological treatment (PSIQ2)**
0 No 1 Yes
**You do physical exercises (ATIVFIS)**
0 Yes 1 no
**Uses hypoglycemic medications, corticosteroids, beta-blockers, or diuretics (MEDIC2)**
0 No 1 Yes
**You currently smoke (FUMA)**
0 No 1 Yes
**You drink alcohol (BEBE)**

0 No 1 Yes
**You have periodontal disease (PERIO2DIC)**
0 No 1 Yes
**Weight (PESO)**
Describe
**Height (ALTURA)**
Describe
**Body Mass Index (IMC)**
Describe
**Systolic Blood Pressure (PASIS)**
Describe
**Diastolic Blood Pressure (PADIAS)**
Describe
**Systolic / Diastolic Blood Pressure (PASISDIA)**
Describe SYSBP/DIASBP
**Blood pressure criteria IDF - (PAIDF) (MetS IDF, AACE, NCEP and IDF/AHA/NHLBI ≥ 130/85)**
0 No 1 Yes
**Altered blood pressure or treatment for sistemic arterial hypertension (SAH) IDF (PAIDF 2)**
0 No (PAIDF and HYPERT =0) 1 Yes (PAIDF and/or HYPERT=1)
**Blood pressure criteria WHO/EGIR- (MetS WHO and EGIR ≥140/90) - (PAWHO)**
0 No 1 Yes
**Altered blood pressure or use of antihypertensive drugs (with SAH) criteria WHO- (PAWHO2)**

0 No (PAWHO and/or HIPERT=0)   1 YES (PAWHO and/or HIPERT=1)
**METS Barbosa 2006 criteria (SMBARB)**
Sum of number of changed items (maximun 5 minimum 0) (GLIIDF2+CABARB+HDLIDF+TRIGIDF+PAIDF2)
**METS Barbosa 2006 (SMBARB2)**
0 No   1 Yes
**METS AHA (SMAHA)**
Sum of number of changed items (maximum 5 minimun 0) (GLIIDF2+CAIDF+HDLIDF+TRIGIDF+PAIDF2)
**Dichotomized METS AHA** SMAHA≥3 **(SMAHA2)**
0 No   1 Yes
**METS NCEP (SMNCEP)**
Sum of number of changed items (maximum 5 minimum 0) (GLIIDF2+CANCEP+HDLIDF+TRIGIDF+PAIDF2)
**Dichotomized NCEP METS** (SMNCEP≥3) **(SMNCEP2)**
0 No   1 Yes
**METS AACE (SMAACE)**
Sum of criteria only (Maximum 4) (HDLIDF+TRIGIDF+PAIDF+GLIAACE)
**Dichotomized METS AACE - ALTORISCORES (mandatory)+ SMAACE ≥2 (SMAACE2)**
0 No   1 Yes
**METS EGIR (SMEGIR)**
Sum of number of changed items (maximum 4 minimum 0) (CAEGIR+HDLEGIR+TRIGIDF+PAWHO)
**METS EGIR dichotomized** GLIWHO (Mandatory)+ SMEGIR ≥ 2 – DIABDIC **(SMEGIR2)**
0 No   1 Yes
**METS IDF (SMIDF)**

Sum of number of changed items (maximum 4 minimum 0) (GLIIDF2+HDLIDF+TRIGIDF+PAIDF2)
**METS dichotomized IDF CAIDF (required) + SMIDF ≥2 (SMIDF2)**
0 No 1 Yes
**METS WHO (SMWHO)**
Sum of number of changed items (maximum 4 minimum 0) (IMC +HDLWHO+TRIGIDF+PAWHO2)
**METS WHO criteria (SMWHO1)**
Sum of number of changed items (maximum 5 minimum 0) (IMC +HDLWHO+TRIGIDF+PAWHO2+ MICROAL)
**METS WHO dichotomized** GLIWHO (required) + SMWHO ≥ 2 (**SMWHO2**)
0 No 1 Yes
**Sum of individuals with MetS in 5 different criteria without WHO (SMAHA2, SMAACE2+SMNCEP2+SMIDF2+SMEGIR2) (SOMA5CRIT)**
Number of positive criteria (minimum 0 maximum 5)
**Sum of individuals with MetS in 6 different criteria with WHO (SMAHA2, SMAACE2+ SMNCEP2+ SMIDF2+ SMEGIR2+ SMWHO2 (SOMA6CRIT)**
Number of positive criteria (minimum 0 maximum 5)
**Group of individuals who have MetS in all 6 criteria including WHO (AGRUP)**
O No 1 Yes
**Group of individuals who have MetS in 05 criteria except WHO (BGRUP)**
O No 1 Yes
**Group of individuals with MetS from 1 to 4 criteria except WHO (CGRUP)**
O No 1 Yes
**Group of individuals who do not have MetS by any criteria (DGRUP)**
O No 1 Yes
**Waist Circumference (CA)**

Describe
**Waist Circumference EGIR (CAEGIR)**
0 <94 men and <80 women 1 ≥94 men and ≥80 women
**Waist Circumference NCEP (CANCEP)**
0 <102 men and <88 women 1 ≥102 men and ≥88 women
**Waist Circumference IDF (CAIDF) (mets IDF and IDF/AHA/NHLBI)**
0 <90 men and <80 women 1 ≥90 men and ≥80 women
**Waist Circumference BARBOSA (CABARB) (Mets Barbosa)**
0 <88 men and <84 women 1 ≥88 men and ≥84 women
**Fasting Blood Glucose (HGT)**
Describe
**Triglycerides (TRIGL)**
Describe
**HDL-C (HDL)**
Describe
**HDL-C WHO (HDLWHO)**
0 ≥ 35 men and ≥39 women 1 < 35 men and <39 women
**HDL-C IDF (HDLIDF) (serves AACE, NCEP, IDF and IDF/AHA/NHLBI)**
0 ≥ 40 men and ≥50 women 1 <40 men and < 50 women
**HDL-C EGIR (HDLEGIR)**
0 ≥ 39 men and women 1 < 39 men and women
**BMI WHO (IMCWHO)**

0 < 30 KG/m^2 1 ≥ 30KG/m^2
**Glycemia WHO (Mets WHO and EGIR) (GLIWHO)**
0 <110 mg/dl 1 ≥110mg/dl
**IDF Glycemia (Mets NCEP, IDF , IDF/AHA/NHLBI) (GLIIDF)**
0 <100 mg/dl 1 ≥100mg/dl
**Altered blood glucose or treatment for diabetes IDF (GLIDF2)**
0 no (GLIIDF and DIAB =0) 1 YES (GLIIDF and/or DIAB=1)
**Glycemia AACE 110-125 (GLIAACE)**
0 <110 or >125 1 110 to 125
**Triglycerides IDF (TRIGIDF) (Mets all criteria)**
0 <150 mg/dl 1≥150mg/dl
**Presence of Acanthosis Nigricans (ACANNIGRI)**
0 No 1 Yes
**You have nonalcoholic liver disease (DOHEPALC)**
0 No 1 Yes
**Have obstructive sleep apnea (APNOBST2)**
0 No 1 Yes
**Burnout Syndrome (BSDIC)**
0 no 1 yes
**Fasting Insulin (INSJEJ)**
Describe
**Dichotomized SB (Ramirez Criteria) (BSDIC)**

0 no 1 yes
**METS-NCEP 2001 (SM2001)**
number of criteria by NCEP 2006
**METS dichotomized NCEP 2001 (SMDIC2001)**
0 no 1 yes
**BMI AACE (IMCAACE)**
0 <25KG/m2 1 ≥ 25KG/m2
**High-risk insulin resistance (ALTORISCRES)**
0 No 1 YES
**Insulin resistance (INSURES)**
0 No 1 Yes
