# Supplementary material for: Diagnosis of metabolic syndrome in nursing professionals: An accuracy study
Source: PLoS One. 2024 Jun 10;19(6):e0295985. doi: 10.1371/journal.pone.0295985 (PMC11164393; doi:10.1371/journal.pone.0295985)
Supplement: S3 File — (PDF) [file pone.0295985.s003.pdf]

```
  ___  ____  ____  ____  ____(R)
 /__    /   ____/   /   ____/
___/   /   /___/   /   /___/
  Statistics/Data Analysis
```

User: dicotomização SM parte 1{space -2}

```
  ___  ____  ____  ____  ____ (R)
 /__    /   ____/   /   ____/
___/   /   /___/   /   /___/   11.2   Copyright 1985-2009 StataCorp LP
  Statistics/Data Analysis            StataCorp
                                      4905 Lakeway Drive
     Special Edition                  College Station, Texas 77845 USA
                                      800-STATA-PC        http://www.stata.com
                                      979-696-4600        stata@stata.com
                                      979-696-4601 (fax)

Single-user Stata license expires 31 Dec 9999:
       Serial number:  71606281563
         Licensed to:  STATAForAll
                       STATA

Notes:
      1.  (/m# option or -set memory-) 50.00 MB allocated to data
      2.  (/v# option or -set maxvar-) 5000 maximum variables
```

1 . use "C:\Users\enfae\Documents\síndrome metabólica\Projeto 2021\Banco de dados SM Magno\ban

2 . tab idad2 sex, row col chi

| Key |
|---|
| *frequency*<br>*row percentage*<br>*column percentage* |

| idad2 | sex 0 | 1 | Total |
|---|---|---|---|
| 0 | 75<br>12.95<br>55.15 | 504<br>87.05<br>51.69 | 579<br>100.00<br>52.12 |
| 1 | 61<br>11.47<br>44.85 | 471<br>88.53<br>48.31 | 532<br>100.00<br>47.88 |
| Total | 136<br>12.24<br>100.00 | 975<br>87.76<br>100.00 | 1,111<br>100.00<br>100.00 |

Pearson chi2(1) =   0.5708   Pr = 0.450

3 . tab  cor2 sex, row col chi

| Key |
|---|
| *frequency*<br>*row percentage*<br>*column percentage* |

| cor2 | sex 0 | 1 | Total |
|---|---|---|---|
| 0 | 38<br>13.57<br>27.94 | 242<br>86.43<br>24.82 | 280<br>100.00<br>25.20 |
| 1 | 98<br>11.79<br>72.06 | 733<br>88.21<br>75.18 | 831<br>100.00<br>74.80 |
| Total | 136<br>12.24<br>100.00 | 975<br>87.76<br>100.00 | 1,111<br>100.00<br>100.00 |

Pearson chi2(1) =   0.6166   Pr = 0.432

4 . tab  resid2 sex, row col chi

| Key |
|---|
| *frequency*<br>*row percentage*<br>*column percentage* |

| resid2 | sex 0 | 1 | Total |
|---|---|---|---|
| 0 | 39<br>21.43<br>28.68 | 143<br>78.57<br>14.67 | 182<br>100.00<br>16.38 |
| 1 | 97<br>10.44<br>71.32 | 832<br>89.56<br>85.33 | 929<br>100.00<br>83.62 |
| Total | 136<br>12.24<br>100.00 | 975<br>87.76<br>100.00 | 1,111<br>100.00<br>100.00 |

Pearson chi2(1) =  17.1015   Pr = 0.000

5 . tab estci2  sex, row col chi

| Key |
|---|
| *frequency*<br>*row percentage*<br>*column percentage* |

| estci2 | sex 0 | 1 | Total |
|---|---|---|---|
| 0 | 70<br>11.71<br>51.47 | 528<br>88.29<br>54.15 | 598<br>100.00<br>53.83 |
| 1 | 66<br>12.87<br>48.53 | 447<br>87.13<br>45.85 | 513<br>100.00<br>46.17 |
| Total | 136<br>12.24 | 975<br>87.76 | 1,111<br>100.00 |

| | 100.00 | 100.00 | 100.00 |
|---|---|---|---|

Pearson chi2(1) = 0.3458 Pr = 0.557

```
6 . tab  nfil2 sex, row col chi
```

| Key |
|---|
| *frequency*<br>*row percentage*<br>*column percentage* |

| nfil2 | sex 0 | 1 | Total |
|---|---|---|---|
| 0 | 67<br>14.89<br>49.26 | 383<br>85.11<br>39.28 | 450<br>100.00<br>40.50 |
| 1 | 69<br>10.44<br>50.74 | 592<br>89.56<br>60.72 | 661<br>100.00<br>59.50 |
| Total | 136<br>12.24<br>100.00 | 975<br>87.76<br>100.00 | 1,111<br>100.00<br>100.00 |

Pearson chi2(1) = 4.9356 Pr = 0.026

```
7 . tab  nfildic sex, row col chi
```

| Key |
|---|
| *frequency*<br>*row percentage*<br>*column percentage* |

| nfildic | sex 0 | 1 | Total |
|---|---|---|---|
| 0 | 122<br>12.76<br>89.71 | 834<br>87.24<br>85.54 | 956<br>100.00<br>86.05 |
| 1 | 14<br>9.03<br>10.29 | 141<br>90.97<br>14.46 | 155<br>100.00<br>13.95 |
| Total | 136<br>12.24<br>100.00 | 975<br>87.76<br>100.00 | 1,111<br>100.00<br>100.00 |

Pearson chi2(1) = 1.7266 Pr = 0.189

```
8 . tab   prof sex, row col chi
```

| Key |
|---|
| *frequency* |
| *row percentage* |
| *column percentage* |

| prof | sex 0 | 1 | Total |
|---|---|---|---|
| 0 | 64<br>14.16<br>47.06 | 388<br>85.84<br>39.79 | 452<br>100.00<br>40.68 |
| 1 | 72<br>10.93<br>52.94 | 587<br>89.07<br>60.21 | 659<br>100.00<br>59.32 |
| Total | 136<br>12.24<br>100.00 | 975<br>87.76<br>100.00 | 1,111<br>100.00<br>100.00 |

Pearson chi2(1) =   2.6096   Pr = 0.106

```
9 . tab  renda2  sex, row col chi
```

| Key |
|---|
| *frequency* |
| *row percentage* |
| *column percentage* |

| renda2 | sex 0 | 1 | Total |
|---|---|---|---|
| 0 | 83<br>13.86<br>61.03 | 516<br>86.14<br>52.92 | 599<br>100.00<br>53.92 |
| 1 | 53<br>10.35<br>38.97 | 459<br>89.65<br>47.08 | 512<br>100.00<br>46.08 |
| Total | 136<br>12.24<br>100.00 | 975<br>87.76<br>100.00 | 1,111<br>100.00<br>100.00 |

Pearson chi2(1) =   3.1565   Pr = 0.076

```
10 . tab  fuma sex, row col chi
```

| Key |
|---|
| *frequency* |
| *row percentage* |
| *column percentage* |

| fuma | sex 0 | sex 1 | Total |
|---|---|---|---|
| 0 | 95<br>9.71<br>69.85 | 883<br>90.29<br>90.56 | 978<br>100.00<br>88.03 |
| 1 | 41<br>30.83<br>30.15 | 92<br>69.17<br>9.44 | 133<br>100.00<br>11.97 |
| Total | 136<br>12.24<br>100.00 | 975<br>87.76<br>100.00 | 1,111<br>100.00<br>100.00 |

Pearson chi2(1) = 48.5821 Pr = 0.000

```
11 . tab bebe2 sex, row col chi
```

| Key |
|---|
| *frequency*<br>*row percentage*<br>*column percentage* |

| bebe2 | sex 0 | sex 1 | Total |
|---|---|---|---|
| 0 | 33<br>8.11<br>24.26 | 374<br>91.89<br>38.36 | 407<br>100.00<br>36.63 |
| 1 | 103<br>14.63<br>75.74 | 601<br>85.37<br>61.64 | 704<br>100.00<br>63.37 |
| Total | 136<br>12.24<br>100.00 | 975<br>87.76<br>100.00 | 1,111<br>100.00<br>100.00 |

Pearson chi2(1) = 10.2135 Pr = 0.001

```
12 . tab  atvfis sex, row col chi
```

| Key |
|---|
| *frequency*<br>*row percentage*<br>*column percentage* |

| atvfis | sex 0 | sex 1 | Total |
|---|---|---|---|
| 0 | 85<br>13.49<br>62.50 | 545<br>86.51<br>55.90 | 630<br>100.00<br>56.71 |
| 1 | 51<br>10.60<br>37.50 | 430<br>89.40<br>44.10 | 481<br>100.00<br>43.29 |
| Total | 136<br>12.24 | 975<br>87.76 | 1,111<br>100.00 |

```
           |     100.00     100.00 |     100.00

          Pearson chi2(1) =   2.1193   Pr = 0.145

13 . gen peso5g= peso

14 . replace peso5g=1 if peso5g>=41& peso5g<56
   (138 real changes made)

15 . replace peso5g=2 if peso5g>=56& peso5g<71
   (504 real changes made)

16 . replace peso5g=3 if peso5g>=71& peso5g<86
   (316 real changes made)

17 . replace peso5g=4 if peso5g>=86& peso5g<101
   (123 real changes made)

18 . replace peso5g=5 if peso5g>=101
   (30 real changes made)

19 . tab peso5g
```

| peso5g | Freq. | Percent | Cum. |
|---|---|---|---|
| 1 | 138 | 12.42 | 12.42 |
| 2 | 504 | 45.36 | 57.79 |
| 3 | 316 | 28.44 | 86.23 |
| 4 | 123 | 11.07 | 97.30 |
| 5 | 30 | 2.70 | 100.00 |
| Total | 1,111 | 100.00 | |

```
20 . gen alt2g= altura

21 . replace alt2g=1 if alt2g>=1.38&  alt2g<1.48
   (9 real changes made)

22 . replace alt2g=2 if alt2g>=1.48&  alt2g<1.58
   (249 real changes made)

23 . replace alt2g=3 if alt2g>=1.58&  alt2g<1.68
   (601 real changes made)

24 . replace alt2g=4 if alt2g>=1.68&  alt2g<1.78
   (204 real changes made)

25 . replace alt2g=5 if alt2g>=1.78
   (1102 real changes made)

26 . tab alt2g
```

| alt2g | Freq. | Percent | Cum. |
|---|---|---|---|
| 1 | 9 | 0.81 | 0.81 |
| 5 | 1,102 | 99.19 | 100.00 |
| Total | 1,111 | 100.00 | |

```
27 . gen altura2g= altura

28 . replace  altura2g=5 if  altura2g>=1.78
   (48 real changes made)

29 . replace  altura2g=1 if alt2g>=1.38&   altura2g<1.48
   (0 real changes made)

30 . replace  altura2g=1 if  altura2g>=1.38&   altura2g<1.48
   (9 real changes made)

31 . replace  altura2g=2 if  altura2g>=1.48&   altura2g<1.58
   (249 real changes made)

32 . replace  altura2g=3 if  altura2g>=1.58&   altura2g<1.68
   (601 real changes made)

33 . replace  altura2g=4 if  altura2g>=1.68&   altura2g<1.78
   (204 real changes made)

34 . tab altura2g
```

| altura2g | Freq. | Percent | Cum. |
|---:|---:|---:|---:|
| 1 | 9 | 0.81 | 0.81 |
| 2 | 249 | 22.41 | 23.22 |
| 3 | 601 | 54.10 | 77.32 |
| 4 | 204 | 18.36 | 95.68 |
| 5 | 48 | 4.32 | 100.00 |
| Total | 1,111 | 100.00 | |

```
35 . gen ca5g= ca

36 . replace  ca5g =5 if   ca5g>=114,5
   option 5 not allowed
   r(198);

37 . replace ca5g=5 if  ca5g>=114,5
   option 5 not allowed
   r(198);

38 . replace ca5g=5 if ca5g>=114.5
   (32 real changes made)

39 . replace ca5g=1 if ca5g>=34,5& ca5g<54,5
   invalid '5'
   r(198);

40 . replace ca5g=1 if ca5g>=34,5& ca5g<54.5
   option 5 not allowed
   r(198);

41 . replace ca5g=1 if ca5g>=34.5 & ca5g<54.5
   (8 real changes made)
```

```
42 . replace ca5g=2 if ca5g>=54.5 & ca5g<74.5
   (219 real changes made)

43 . replace ca5g=3 if ca5g>=74.5 & ca5g<94.5
   (568 real changes made)

44 . replace ca5g=4 if ca5g>=94.5 & ca5g<114.5
   (284 real changes made)

45 . tab ca5g
```

| ca5g | Freq. | Percent | Cum. |
|---:|---:|---:|---:|
| 1 | 8 | 0.72 | 0.72 |
| 2 | 219 | 19.71 | 20.43 |
| 3 | 568 | 51.13 | 71.56 |
| 4 | 284 | 25.56 | 97.12 |
| 5 | 32 | 2.88 | 100.00 |
| Total | 1,111 | 100.00 | |

```
46 . tab smaha2 sex, row col chi
```

| Key |
|---|
| *frequency*<br>*row percentage*<br>*column percentage* |

| | sex | | |
|---:|---:|---:|---:|
| smaha2 | 0 | 1 | Total |
| 0 | 92<br>12.04<br>67.65 | 672<br>87.96<br>68.92 | 764<br>100.00<br>68.77 |
| 1 | 44<br>12.68<br>32.35 | 303<br>87.32<br>31.08 | 347<br>100.00<br>31.23 |
| Total | 136<br>12.24<br>100.00 | 975<br>87.76<br>100.00 | 1,111<br>100.00<br>100.00 |

```
          Pearson chi2(1) =   0.0905   Pr = 0.764

47 . tab  smidf2 sex, row col chi
```

| Key |
|---|
| *frequency*<br>*row percentage*<br>*column percentage* |

| smidf2 | sex 0 | 1 | Total |
|---|---|---|---|
| 0 | 93<br>11.91<br>68.38 | 688<br>88.09<br>70.56 | 781<br>100.00<br>70.30 |
| 1 | 43<br>13.03<br>31.62 | 287<br>86.97<br>29.44 | 330<br>100.00<br>29.70 |
| Total | 136<br>12.24<br>100.00 | 975<br>87.76<br>100.00 | 1,111<br>100.00<br>100.00 |

Pearson chi2(1) =   0.2721   Pr = 0.602

48 . tab   smncep2  sex, row col chi

| Key |
|---|
| *frequency*<br>*row percentage*<br>*column percentage* |

| smncep2 | sex 0 | 1 | Total |
|---|---|---|---|
| 0 | 97<br>11.81<br>71.32 | 724<br>88.19<br>74.26 | 821<br>100.00<br>73.90 |
| 1 | 39<br>13.45<br>28.68 | 251<br>86.55<br>25.74 | 290<br>100.00<br>26.10 |
| Total | 136<br>12.24<br>100.00 | 975<br>87.76<br>100.00 | 1,111<br>100.00<br>100.00 |

Pearson chi2(1) =   0.5322   Pr = 0.466

49 . tab  smbarb2  sex, row col chi

| Key |
|---|
| *frequency*<br>*row percentage*<br>*column percentage* |

| smbarb2 | sex 0 | 1 | Total |
|---|---|---|---|
| 0 | 92<br>11.68<br>67.65 | 696<br>88.32<br>71.38 | 788<br>100.00<br>70.93 |
| 1 | 44<br>13.62<br>32.35 | 279<br>86.38<br>28.62 | 323<br>100.00<br>29.07 |
| Total | 136<br>12.24 | 975<br>87.76 | 1,111<br>100.00 |

```
            |     100.00     100.00 |     100.00

          Pearson chi2(1) =   0.8085   Pr = 0.369

50 . tab   smegir2  sex, row col chi

  +-------------------+
  | Key               |
  |-------------------|
  |     frequency     |
  |  row percentage   |
  | column percentage |
  +-------------------+

           |          sex
   smegir2 |         0          1 |     Total
-----------+----------------------+----------
         0 |       123        936 |     1,059 
           |     11.61      88.39 |    100.00 
           |     90.44      96.00 |     95.32 
-----------+----------------------+----------
         1 |        13         39 |        52 
           |     25.00      75.00 |    100.00 
           |      9.56       4.00 |      4.68 
-----------+----------------------+----------
     Total |       136        975 |     1,111 
           |     12.24      87.76 |    100.00 
           |    100.00     100.00 |    100.00 

          Pearson chi2(1) =   8.2665   Pr = 0.004

51 . tab  smaace2 sex, row col chi

  +-------------------+
  | Key               |
  |-------------------|
  |     frequency     |
  |  row percentage   |
  | column percentage |
  +-------------------+

           |          sex
   smaace2 |         0          1 |     Total
-----------+----------------------+----------
         0 |       101        862 |       963 
           |     10.49      89.51 |    100.00 
           |     74.26      88.41 |     86.68 
-----------+----------------------+----------
         1 |        35        113 |       148 
           |     23.65      76.35 |    100.00 
           |     25.74      11.59 |     13.32 
-----------+----------------------+----------
     Total |       136        975 |     1,111 
           |     12.24      87.76 |    100.00 
           |    100.00     100.00 |    100.00 

          Pearson chi2(1) =  20.6828   Pr = 0.000

52 . save "C:\Users\enfae\Documents\síndrome metabólica\Projeto 2021\calculos STATA\calculos tal
   file C:\Users\enfae\Documents\síndrome metabólica\Projeto 2021\calculos STATA\calculos tabel

53 . save "C:\Users\enfae\Documents\síndrome metabólica\Projeto 2021\Banco de dados SM Magno\bar
   file C:\Users\enfae\Documents\síndrome metabólica\Projeto 2021\Banco de dados SM Magno\banco

54 .
```

```
  ___  ____  ____  ____  ____(R)
 /__    /   ____/   /   ____/
___/   /   /___/   /   /___/
  Statistics/Data Analysis
```

User: calculo tab 2 carct clinicas{space -6}

```
  ___  ____  ____  ____  ____ (R)
 /__    /   ____/   /   ____/
___/   /   /___/   /   /___/   11.2
  Statistics/Data Analysis
```

*Special Edition*

Copyright 1985-2009 StataCorp LP
StataCorp
4905 Lakeway Drive
College Station, Texas 77845 USA
800-STATA-PC        http://www.stata.com
979-696-4600        stata@stata.com
979-696-4601 (fax)

Single-user Stata license expires 31 Dec 9999:
Serial number: 71606281563
Licensed to: STATAForAll
STATA

Notes:
1. (/m# option or -set memory-) 50.00 MB allocated to data
2. (/v# option or -set maxvar-) 5000 maximum variables

1 . use "C:\Users\enfae\Documents\síndrome metabólica\Projeto 2021\Banco de dados SM Magno\

2 . bysort sex: sum pasis

-> sex = 0

| Variable | Obs | Mean | Std. Dev. | Min | Max |
|---|---|---|---|---|---|
| pasis | 136 | 123.7059 | 14.09445 | 100 | 160 |

-> sex = 1

| Variable | Obs | Mean | Std. Dev. | Min | Max |
|---|---|---|---|---|---|
| pasis | 975 | 116.5805 | 15.44186 | 80 | 180 |

3 . bysort sex: sum  padias

-> sex = 0

| Variable | Obs | Mean | Std. Dev. | Min | Max |
|---|---|---|---|---|---|
| padias | 136 | 82.55147 | 9.417729 | 60 | 100 |

-> sex = 1

| Variable | Obs | Mean | Std. Dev. | Min | Max |
|---|---|---|---|---|---|
| padias | 975 | 77.02667 | 10.61 | 50 | 120 |

4 . bysort sex: sum ca

-> sex = 0

| Variable | Obs | Mean | Std. Dev. | Min | Max |
|---|---|---|---|---|---|
| ca | 136 | 89.60294 | 15.71389 | 34.5 | 129 |

-> sex = 1

| Variable | Obs | Mean | Std. Dev. | Min | Max |
|---|---|---|---|---|---|
| ca | 975 | 86.33487 | 14.11161 | 38 | 143 |

5 . bysort sex: sum hgt

-> sex = 0

| Variable | Obs | Mean | Std. Dev. | Min | Max |
|---|---|---|---|---|---|
| hgt | 136 | 86.19191 | 16.08583 | 49 | 124 |

-> sex = 1

| Variable | Obs | Mean | Std. Dev. | Min | Max |
|---|---|---|---|---|---|
| hgt | 975 | 84.48092 | 18.04765 | 41 | 220 |

6 . bysort sex: sum trigl

-> sex = 0

| Variable | Obs | Mean | Std. Dev. | Min | Max |
|---|---|---|---|---|---|
| trigl | 136 | 148.1757 | 67.27139 | 30 | 486 |

-> sex = 1

| Variable | Obs | Mean | Std. Dev. | Min | Max |
|---|---|---|---|---|---|
| trigl | 975 | 132.4146 | 55.24067 | 26 | 362 |

7 . bysort sex: sum hdl

-> sex = 0

| Variable | Obs | Mean | Std. Dev. | Min | Max |
|---|---|---|---|---|---|
| hdl | 136 | 57.72794 | 27.02426 | 19 | 207 |

-> sex = 1

| Variable | Obs | Mean | Std. Dev. | Min | Max |
|---|---|---|---|---|---|
| hdl | 975 | 58.80178 | 31.30097 | 18 | 270 |

8 . bysort sex: sum imc

-> sex = 0

| Variable | Obs | Mean | Std. Dev. | Min | Max |
|---|---|---|---|---|---|
| imc | 136 | 27.55801 | 4.505698 | 19.72318 | 41.01563 |

-> sex = 1

| Variable | Obs | Mean | Std. Dev. | Min | Max |
|---|---|---|---|---|---|
| imc | 975 | 26.18554 | 4.642457 | 15.58957 | 43.75 |

9 . tab sex

| sex | Freq. | Percent | Cum. |
|---|---|---|---|
| 0 | 136 | 12.24 | 12.24 |
| 1 | 975 | 87.76 | 100.00 |
| Total | 1,111 | 100.00 | |

10 . ttest pasis, by sex
option by incorrectly specified
r(198);

11 . ttest pasis, by (sex)

Two-sample t test with equal variances

| Group | Obs | Mean | Std. Err. | Std. Dev. | [95% Conf. | Interval] |
|---|---|---|---|---|---|---|
| 0 | 136 | 123.7059 | 1.208589 | 14.09445 | 121.3157 | 126.0961 |
| 1 | 975 | 116.5805 | .4945354 | 15.44186 | 115.61 | 117.551 |
| combined | 1111 | 117.4527 | .4636714 | 15.45494 | 116.543 | 118.3625 |
| diff | | 7.12537 | 1.399032 | | 4.38032 | 9.870419 |

```
    diff = mean(0) - mean(1)                                      t =   5.0931
Ho: diff = 0                                     degrees of freedom =     1109

    Ha: diff < 0                 Ha: diff != 0                 Ha: diff > 0
 Pr(T < t) = 1.0000         Pr(|T| > |t|) = 0.0000          Pr(T > t) = 0.0000
```

12 . ttest hgt, by (sex)

Two-sample t test with equal variances

| Group | Obs | Mean | Std. Err. | Std. Dev. | [95% Conf. | Interval] |
|---|---|---|---|---|---|---|
| 0 | 136 | 86.19191 | 1.379349 | 16.08583 | 83.46398 | 88.91984 |
| 1 | 975 | 84.48092 | .5779874 | 18.04765 | 83.34668 | 85.61517 |
| combined | 1111 | 84.69037 | .5346625 | 17.82119 | 83.64131 | 85.73943 |
| diff | | 1.710989 | 1.631182 | | -1.489562 | 4.911539 |

```
    diff = mean(0) - mean(1)                                      t =   1.0489
Ho: diff = 0                                     degrees of freedom =     1109

    Ha: diff < 0                 Ha: diff != 0                 Ha: diff > 0
 Pr(T < t) = 0.8528         Pr(|T| > |t|) = 0.2944          Pr(T > t) = 0.1472
```

13 . ttest  trigl, by (sex)

Two-sample t test with equal variances

| Group | Obs | Mean | Std. Err. | Std. Dev. | [95% Conf. | Interval] |
|---|---|---|---|---|---|---|
| 0 | 136 | 148.1757 | 5.768474 | 67.27139 | 136.7675 | 159.584 |
| 1 | 975 | 132.4146 | 1.769117 | 55.24067 | 128.9428 | 135.8863 |
| combined | 1111 | 134.3439 | 1.711599 | 57.05044 | 130.9856 | 137.7023 |
| diff | | 15.76117 | 5.202961 | | 5.552414 | 25.96993 |

```
    diff = mean(0) - mean(1)                                      t =   3.0293
Ho: diff = 0                                     degrees of freedom =     1109

    Ha: diff < 0                 Ha: diff != 0                 Ha: diff > 0
 Pr(T < t) = 0.9987         Pr(|T| > |t|) = 0.0025          Pr(T > t) = 0.0013
```

14 . ttest   hdl, by (sex)

Two-sample t test with equal variances

| Group | Obs | Mean | Std. Err. | Std. Dev. | [95% Conf. | Interval] |
|---|---|---|---|---|---|---|
| 0 | 136 | 57.72794 | 2.317311 | 27.02426 | 53.14501 | 62.31087 |
| 1 | 975 | 58.80178 | 1.002433 | 31.30097 | 56.83461 | 60.76896 |
| combined | 1111 | 58.67033 | .9240534 | 30.80024 | 56.85724 | 60.48342 |
| diff | | -1.073843 | 2.820374 | | -6.607714 | 4.460028 |

diff = mean(0) - mean(1)                                  t =  -0.3807
Ho: diff = 0                                 degrees of freedom =     1109

Ha: diff < 0                 Ha: diff != 0                 Ha: diff > 0
Pr(T < t) = 0.3517         Pr(|T| > |t|) = 0.7035          Pr(T > t) = 0.6483

15 . ttest  imc, by (sex)

Two-sample t test with equal variances

| Group | Obs | Mean | Std. Err. | Std. Dev. | [95% Conf. | Interval] |
|---|---|---|---|---|---|---|
| 0 | 136 | 27.55801 | .3863604 | 4.505698 | 26.79391 | 28.32211 |
| 1 | 975 | 26.18554 | .1486776 | 4.642457 | 25.89378 | 26.47731 |
| combined | 1111 | 26.35355 | .1393807 | 4.645791 | 26.08007 | 26.62703 |
| diff | | 1.372469 | .4234413 | | .5416327 | 2.203306 |

diff = mean(0) - mean(1)                                  t =   3.2412
Ho: diff = 0                                 degrees of freedom =     1109

Ha: diff < 0                 Ha: diff != 0                 Ha: diff > 0
Pr(T < t) = 0.9994         Pr(|T| > |t|) = 0.0012          Pr(T > t) = 0.0006

16 . ttest  ca, by (sex)

Two-sample t test with equal variances

| Group | Obs | Mean | Std. Err. | Std. Dev. | [95% Conf. | Interval] |
|---|---|---|---|---|---|---|
| 0 | 136 | 89.60294 | 1.347455 | 15.71389 | 86.93809 | 92.26779 |
| 1 | 975 | 86.33487 | .4519332 | 14.11161 | 85.448 | 87.22175 |
| combined | 1111 | 86.73492 | .4305175 | 14.34987 | 85.8902 | 87.57964 |
| diff | | 3.268069 | 1.310432 | | .6968642 | 5.839275 |

diff = mean(0) - mean(1)                                  t =   2.4939
Ho: diff = 0                                 degrees of freedom =     1109

Ha: diff < 0                 Ha: diff != 0                 Ha: diff > 0
Pr(T < t) = 0.9936         Pr(|T| > |t|) = 0.0128          Pr(T > t) = 0.0064

17 . ttest  padias, by (sex)

Two-sample t test with equal variances

| Group | Obs | Mean | Std. Err. | Std. Dev. | [95% Conf. | Interval] |
|---|---|---|---|---|---|---|
| 0 | 136 | 82.55147 | .8075636 | 9.417729 | 80.95436 | 84.14858 |
| 1 | 975 | 77.02667 | .3397921 | 10.61 | 76.35986 | 77.69348 |
| combined | 1111 | 77.70297 | .3187066 | 10.62302 | 77.07763 | 78.32831 |
| diff | | 5.524804 | .9585618 | | 3.644005 | 7.405603 |

diff = mean(0) - mean(1)                                  t =   5.7636
Ho: diff = 0                                 degrees of freedom =     1109

```
        Ha: diff < 0                 Ha: diff != 0                 Ha: diff > 0
     Pr(T < t) = 1.0000         Pr(|T| > |t|) = 0.0000          Pr(T > t) = 0.0000

18 .
```

```
                                             ___  ____  ____  ____  ____(R)
                                            /__    /   ____/   /   ____/
                                           ___/   /   /___/   /   /___/
                                             Statistics/Data Analysis

                                 User: calculo tab 2 carct clinicas{space -6}
```

```
  ___  ____  ____  ____  ____ (R)
 /__    /   ____/   /   ____/
___/   /   /___/   /   /___/   11.2   Copyright 1985-2009 StataCorp LP
  Statistics/Data Analysis            StataCorp
                                      4905 Lakeway Drive
     Special Edition                  College Station, Texas 77845 USA
                                      800-STATA-PC        http://www.stata.com
                                      979-696-4600        stata@stata.com
                                      979-696-4601 (fax)

Single-user Stata license expires 31 Dec 9999:
       Serial number:  71606281563
         Licensed to:  STATAForAll
                       STATA

Notes:
      1.  (/m# option or -set memory-) 50.00 MB allocated to data
      2.  (/v# option or -set maxvar-) 5000 maximum variables
```

```
1 . use "C:\Users\enfae\Documents\síndrome metabólica\Projeto 2021\Banco de dados SM Magno\

2 . tab glidf  sex, row col chi
```

| Key |
|---|
| *frequency* |
| *row percentage* |
| *column percentage* |

| glidf | sex 0 | 1 | Total |
|---|---|---|---|
| 0 | 109<br>11.78<br>80.15 | 816<br>88.22<br>83.69 | 925<br>100.00<br>83.26 |
| 1 | 27<br>14.52<br>19.85 | 159<br>85.48<br>16.31 | 186<br>100.00<br>16.74 |
| Total | 136<br>12.24<br>100.00 | 975<br>87.76<br>100.00 | 1,111<br>100.00<br>100.00 |

Pearson chi2(1) =   1.0762   Pr = 0.300

```
3 . tab gliwho sex, row col chi
```

| Key |
|---|
| *frequency* |
| *row percentage* |
| *column percentage* |

| gliwho | sex 0 | 1 | Total |
|---|---|---|---|
| 0 | 120<br>11.67<br>88.24 | 908<br>88.33<br>93.13 | 1,028<br>100.00<br>92.53 |
| 1 | 16<br>19.28<br>11.76 | 67<br>80.72<br>6.87 | 83<br>100.00<br>7.47 |
| Total | 136<br>12.24 | 975<br>87.76 | 1,111<br>100.00 |

| | | | |
|---|---|---|---|
| | 100.00 | 100.00 | 100.00 |

Pearson chi2(1) =   4.1335   Pr = 0.042

4 . tab caidf sex, row col chi

| Key |
|---|
| *frequency*<br>*row percentage*<br>*column percentage* |

| caidf | sex 0 | 1 | Total |
|---|---|---|---|
| 0 | 65<br>17.38<br>47.79 | 309<br>82.62<br>31.69 | 374<br>100.00<br>33.66 |
| 1 | 71<br>9.63<br>52.21 | 666<br>90.37<br>68.31 | 737<br>100.00<br>66.34 |
| Total | 136<br>12.24<br>100.00 | 975<br>87.76<br>100.00 | 1,111<br>100.00<br>100.00 |

Pearson chi2(1) =  13.8570   Pr = 0.000

5 . tab caegir sex, row col chi

| Key |
|---|
| *frequency*<br>*row percentage*<br>*column percentage* |

| caegir | sex 0 | 1 | Total |
|---|---|---|---|
| 0 | 81<br>20.77<br>59.56 | 309<br>79.23<br>31.69 | 390<br>100.00<br>35.10 |
| 1 | 55<br>7.63<br>40.44 | 666<br>92.37<br>68.31 | 721<br>100.00<br>64.90 |
| Total | 136<br>12.24<br>100.00 | 975<br>87.76<br>100.00 | 1,111<br>100.00<br>100.00 |

Pearson chi2(1) =  40.6839   Pr = 0.000

6 . tab cancep sex, row col chi

| Key |
|---|
| *frequency*<br>*row percentage*<br>*column percentage* |

| cancep | sex 0 | 1 | Total |
|---|---|---|---|
| 0 | 96<br>14.72<br>70.59 | 556<br>85.28<br>57.03 | 652<br>100.00<br>58.69 |
| 1 | 40<br>8.71<br>29.41 | 419<br>91.29<br>42.97 | 459<br>100.00<br>41.31 |
| Total | 136<br>12.24<br>100.00 | 975<br>87.76<br>100.00 | 1,111<br>100.00<br>100.00 |

Pearson chi2(1) = 9.0549 Pr = 0.003

7 . tab cabarb sex, row col chi

| Key |
|---|
| *frequency*<br>*row percentage*<br>*column percentage* |

| cabarb | sex 0 | 1 | Total |
|---|---|---|---|
| 0 | 63<br>12.23<br>46.32 | 452<br>87.77<br>46.36 | 515<br>100.00<br>46.35 |
| 1 | 73<br>12.25<br>53.68 | 523<br>87.75<br>53.64 | 596<br>100.00<br>53.65 |
| Total | 136<br>12.24<br>100.00 | 975<br>87.76<br>100.00 | 1,111<br>100.00<br>100.00 |

Pearson chi2(1) = 0.0001 Pr = 0.994

8 . tab hdlidf sex, row col chi

| Key |
|---|
| *frequency*<br>*row percentage*<br>*column percentage* |

| hdlidf | sex 0 | 1 | Total |
|---|---|---|---|
| 0 | 105<br>16.88<br>77.21 | 517<br>83.12<br>53.03 | 622<br>100.00<br>55.99 |
| 1 | 31<br>6.34<br>22.79 | 458<br>93.66<br>46.97 | 489<br>100.00<br>44.01 |
| Total | 136<br>12.24<br>100.00 | 975<br>87.76<br>100.00 | 1,111<br>100.00<br>100.00 |

Pearson chi2(1) = 28.3191 Pr = 0.000

9 . tab hdlegir sex, row col chi

| Key |
|---|
| *frequency*<br>*row percentage*<br>*column percentage* |

| hdlegir | sex 0 | 1 | Total |
|---|---|---|---|
| 0 | 112<br>12.40<br>82.35 | 791<br>87.60<br>81.13 | 903<br>100.00<br>81.28 |
| 1 | 24<br>11.54<br>17.65 | 184<br>88.46<br>18.87 | 208<br>100.00<br>18.72 |
| Total | 136<br>12.24<br>100.00 | 975<br>87.76<br>100.00 | 1,111<br>100.00<br>100.00 |

Pearson chi2(1) =   0.1176   Pr = 0.732

10 . tab paidf sex, row col chi

| Key |
|---|
| *frequency*<br>*row percentage*<br>*column percentage* |

| paidf | sex 0 | 1 | Total |
|---|---|---|---|
| 0 | 99<br>10.83<br>72.79 | 815<br>89.17<br>83.59 | 914<br>100.00<br>82.27 |
| 1 | 37<br>18.78<br>27.21 | 160<br>81.22<br>16.41 | 197<br>100.00<br>17.73 |
| Total | 136<br>12.24<br>100.00 | 975<br>87.76<br>100.00 | 1,111<br>100.00<br>100.00 |

Pearson chi2(1) =   9.5354   Pr = 0.002

11 . tab pawho sex, row col chi

| Key |
|---|
| *frequency*<br>*row percentage*<br>*column percentage* |

| pawho | sex 0 | 1 | Total |
|---|---|---|---|
| 0 | 109<br>11.02<br>80.15 | 880<br>88.98<br>90.26 | 989<br>100.00<br>89.02 |
| 1 | 27<br>22.31<br>19.85 | 94<br>77.69<br>9.64 | 121<br>100.00<br>10.89 |
| 2 | 0<br>0.00<br>0.00 | 1<br>100.00<br>0.10 | 1<br>100.00<br>0.09 |
| Total | 136<br>12.24<br>100.00 | 975<br>87.76<br>100.00 | 1,111<br>100.00<br>100.00 |

Pearson chi2(2) = 12.9378 Pr = 0.002

```
12 . tab trigidf sex, row col chi
```

| Key |
|---|
| *frequency*<br>*row percentage*<br>*column percentage* |

| trigidf | sex 0 | 1 | Total |
|---|---|---|---|
| 0 | 80<br>10.81<br>58.82 | 660<br>89.19<br>67.69 | 740<br>100.00<br>66.61 |
| 1 | 56<br>15.09<br>41.18 | 315<br>84.91<br>32.31 | 371<br>100.00<br>33.39 |
| Total | 136<br>12.24<br>100.00 | 975<br>87.76<br>100.00 | 1,111<br>100.00<br>100.00 |

Pearson chi2(1) = 4.2207 Pr = 0.040

```
13 . save "C:\Users\enfae\Documents\Artigo sobre SM\Artigo revisado plos one\calculos stata t
   file C:\Users\enfae\Documents\Artigo sobre SM\Artigo revisado plos one\calculos stata tab

14 .
```

```
                                               ___  ____  ____  ____  ____(R)
                                              /__    /   ____/   /   ____/
                                             ___/   /   /___/   /   /___/
                                               Statistics/Data Analysis

                                       User: dicotomização SM parte 1{space -2}

  ___  ____  ____  ____  ____ (R)
 /__    /   ____/   /   ____/
___/   /   /___/   /   /___/   11.2   Copyright 1985-2009 StataCorp LP
  Statistics/Data Analysis            StataCorp
                                      4905 Lakeway Drive
     Special Edition                  College Station, Texas 77845 USA
                                      800-STATA-PC        http://www.stata.com
                                      979-696-4600        stata@stata.com
                                      979-696-4601 (fax)

Single-user Stata license expires 31 Dec 9999:
       Serial number:  71606281563
         Licensed to:  STATAForAll
                       STATA

Notes:
      1.  (/m# option or -set memory-) 50.00 MB allocated to data
      2.  (/v# option or -set maxvar-) 5000 maximum variables
```

1 . use "C:\Users\enfae\Documents\síndrome metabólica\Projeto 2021\Banco de dados SM Magno\

2 . ci smbarb2

| Variable | Obs | Mean | Std. Err. | [95% Conf. | Interval] |
|---|---|---|---|---|---|
| smbarb2 | 1111 | .2907291 | .0136298 | .263986 | .3174721 |

3 . ci smaha2

| Variable | Obs | Mean | Std. Err. | [95% Conf. | Interval] |
|---|---|---|---|---|---|
| smaha2 | 1111 | .3123312 | .0139103 | .2850378 | .3396246 |

4 . ci smncep2

| Variable | Obs | Mean | Std. Err. | [95% Conf. | Interval] |
|---|---|---|---|---|---|
| smncep2 | 1111 | .2610261 | .0131824 | .2351608 | .2868914 |

5 . ci smaace2

| Variable | Obs | Mean | Std. Err. | [95% Conf. | Interval] |
|---|---|---|---|---|---|
| smaace2 | 1111 | .1332133 | .0101993 | .1132013 | .1532253 |

6 . ci smegir2

| Variable | Obs | Mean | Std. Err. | [95% Conf. | Interval] |
|---|---|---|---|---|---|
| smegir2 | 1111 | .0468047 | .0063398 | .0343654 | .059244 |

7 . ci smidf2

| Variable | Obs | Mean | Std. Err. | [95% Conf. | Interval] |
|---|---|---|---|---|---|
| smidf2 | 1111 | .2970297 | .0137154 | .2701188 | .3239406 |

8 . ci smidf2 by sort sex
**variable by not found**
r(111);

```
 9 . by sort sex: ci smbarb2
   variable sort not found
   r(111);

10 . bysort sex: ci smbarb2

-------------------------------------------------------------------------------
-> sex = 0

    Variable |        Obs        Mean    Std. Err.       [95% Conf. Interval]
-------------+---------------------------------------------------------------
     smbarb2 |        136    .3235294    .0402638        .2439001    .4031588

-------------------------------------------------------------------------------
-> sex = 1

    Variable |        Obs        Mean    Std. Err.       [95% Conf. Interval]
-------------+---------------------------------------------------------------
     smbarb2 |        975    .2861538    .0144818        .2577347     .314573

11 . bysort sex: ci  smncep2

-------------------------------------------------------------------------------
-> sex = 0

    Variable |        Obs        Mean    Std. Err.       [95% Conf. Interval]
-------------+---------------------------------------------------------------
     smncep2 |        136    .2867647    .0389235        .2097859    .3637435

-------------------------------------------------------------------------------
-> sex = 1

    Variable |        Obs        Mean    Std. Err.       [95% Conf. Interval]
-------------+---------------------------------------------------------------
     smncep2 |        975    .2574359    .0140095        .2299437    .2849281

12 . bysort sex: ci   smegir2

-------------------------------------------------------------------------------
-> sex = 0

    Variable |        Obs        Mean    Std. Err.       [95% Conf. Interval]
-------------+---------------------------------------------------------------
     smegir2 |        136    .0955882    .0253057        .0455413    .1456351

-------------------------------------------------------------------------------
-> sex = 1

    Variable |        Obs        Mean    Std. Err.       [95% Conf. Interval]
-------------+---------------------------------------------------------------
     smegir2 |        975         .04    .0062789        .0276782    .0523218

13 . bysort sex: ci   smidf2

-------------------------------------------------------------------------------
-> sex = 0

    Variable |        Obs        Mean    Std. Err.       [95% Conf. Interval]
-------------+---------------------------------------------------------------
      smidf2 |        136    .3161765    .0400193        .2370305    .3953224

-------------------------------------------------------------------------------
-> sex = 1

    Variable |        Obs        Mean    Std. Err.       [95% Conf. Interval]
-------------+---------------------------------------------------------------
      smidf2 |        975     .294359    .0146033        .2657014    .3230165
```

14 . bysort sex: ci smidf2

-> sex = 0

| Variable | Obs | Mean | Std. Err. | [95% Conf. | Interval] |
|---|---|---|---|---|---|
| smidf2 | 136 | .3161765 | .0400193 | .2370305 | .3953224 |

-> sex = 1

| Variable | Obs | Mean | Std. Err. | [95% Conf. | Interval] |
|---|---|---|---|---|---|
| smidf2 | 975 | .294359 | .0146033 | .2657014 | .3230165 |

15 . bysort sex: ci smaha2

-> sex = 0

| Variable | Obs | Mean | Std. Err. | [95% Conf. | Interval] |
|---|---|---|---|---|---|
| smaha2 | 136 | .3235294 | .0402638 | .2439001 | .4031588 |

-> sex = 1

| Variable | Obs | Mean | Std. Err. | [95% Conf. | Interval] |
|---|---|---|---|---|---|
| smaha2 | 975 | .3107692 | .0148293 | .2816681 | .3398704 |

16 . bysort sex: ci smaace2

-> sex = 0

| Variable | Obs | Mean | Std. Err. | [95% Conf. | Interval] |
|---|---|---|---|---|---|
| smaace2 | 136 | .2573529 | .0376261 | .1829401 | .3317657 |

-> sex = 1

| Variable | Obs | Mean | Std. Err. | [95% Conf. | Interval] |
|---|---|---|---|---|---|
| smaace2 | 975 | .1158974 | .0102567 | .0957696 | .1360253 |

17 .

```
-----------------------------------------------------------------------------------------
------------------------
      name:  <unnamed>
       log:  C:\Users\User\OneDrive\ANALISES\JULITA\Julita.log
  log type:  text
 opened on:   1 Aug 2021, 10:56:43

. do "C:\Users\User\AppData\Local\Temp\STD2f8_000000.tmp"

. **diagti   verdadeiro_positivo falso_negativo falso_positivo verdadeiro_negativo
smbarb2 smaha2
.
. diagti 322 25 1 763
command diagti is unrecognized
r(199);

end of do-file

r(199);

. do "C:\Users\User\AppData\Local\Temp\STD2f8_000000.tmp"

. diagti 322 25 1 763

      True |
   disease |      Test result
    status |      Neg.       Pos. |     Total
-----------+----------------------+----------
    Normal |       763          1 |       764
  Abnormal |        25        322 |       347
-----------+----------------------+----------
     Total |       788        323 |     1,111


                                                            [95% Confidence Interval]
---------------------------------------------------------------------------
Prevalence                           Pr(A)       31%       29%      34.1%
---------------------------------------------------------------------------
Sensitivity                         Pr(+|A)     92.8%     89.5%     95.3%
Specificity                         Pr(-|N)     99.9%     99.3%      100%
ROC area                  (Sens. + Spec.)/2      .963       .95      .977
---------------------------------------------------------------------------
Likelihood ratio (+)     Pr(+|A)/Pr(+|N)       709       100      5028
Likelihood ratio (-)     Pr(-|A)/Pr(-|N)     .0721     .0495      .105
Odds ratio                     LR(+)/LR(-)      9827      1659         .
Positive predictive value           Pr(A|+)     99.7%     98.3%      100%
Negative predictive value           Pr(N|-)     96.8%     95.4%     97.9%
---------------------------------------------------------------------------
```

```
.
. **diagti   verdadeiro_positivo falso_negativo falso_positivo verdadeiro_negativo
ncep2 smaha2
.
. diagti 290 57 0 764

      True |
   disease |      Test result
    status |      Neg.       Pos. |     Total
-----------+----------------------+----------
    Normal |       764          0 |       764
  Abnormal |        57        290 |       347
-----------+----------------------+----------
     Total |       821        290 |     1,111


                                                  [95% Confidence Interval]
---------------------------------------------------------------------------
Prevalence                         Pr(A)       31%       29%      34.1%
---------------------------------------------------------------------------
Sensitivity                      Pr(+|A)     83.6%     79.2%     87.3%
Specificity                      Pr(-|N)      100%     99.5%      100%
ROC area               (Sens. + Spec.)/2      .918      .898      .937
---------------------------------------------------------------------------
Likelihood ratio (+)     Pr(+|A)/Pr(+|N)         .         .         .
Likelihood ratio (-)     Pr(-|A)/Pr(-|N)      .164       .13      .208
Odds ratio                   LR(+)/LR(-)         .      1007         .
Positive predictive value        Pr(A|+)      100%     98.7%      100%
Negative predictive value        Pr(N|-)     93.1%     91.1%     94.7%
---------------------------------------------------------------------------

  Missing values or confidence intervals may be estimated
  using the -sf- or -sf0- options.

.
. **diagti   verdadeiro_positivo falso_negativo falso_positivo verdadeiro_negativo
smaace2 smaha2
.
. diagti 144 203 4 760

      True |
   disease |      Test result
    status |      Neg.       Pos. |     Total
-----------+----------------------+----------
    Normal |       760          4 |       764
  Abnormal |       203        144 |       347
-----------+----------------------+----------
     Total |       963        148 |     1,111
```

```
                                                  [95% Confidence Interval]
---------------------------------------------------------------------------
Prevalence                         Pr(A)       31%       29%      34.1%
---------------------------------------------------------------------------
Sensitivity                      Pr(+|A)     41.5%     36.3%     46.9%
Specificity                      Pr(-|N)     99.5%     98.7%     99.9%
ROC area               (Sens. + Spec.)/2      .705      .679      .731
---------------------------------------------------------------------------
Likelihood ratio (+)     Pr(+|A)/Pr(+|N)      79.3      29.6       212
Likelihood ratio (-)     Pr(-|A)/Pr(-|N)      .588      .538      .643
Odds ratio                   LR(+)/LR(-)       135      51.2       355
Positive predictive value        Pr(A|+)     97.3%     93.2%     99.3%
Negative predictive value        Pr(N|-)     78.9%     76.2%     81.5%
---------------------------------------------------------------------------

.
. **diagti   verdadeiro_positivo falso_negativo falso_positivo verdadeiro_negativo
smegir2 smaha2
.
. diagti 52 295 0 764

      True |
   disease |      Test result
    status |      Neg.       Pos. |     Total
-----------+----------------------+----------
    Normal |       764          0 |       764
  Abnormal |       295         52 |       347
-----------+----------------------+----------
     Total |     1,059         52 |     1,111


                                                  [95% Confidence Interval]
---------------------------------------------------------------------------
Prevalence                         Pr(A)       31%       29%      34.1%
---------------------------------------------------------------------------
Sensitivity                      Pr(+|A)       15%     11.4%     19.2%
Specificity                      Pr(-|N)      100%     99.5%      100%
ROC area               (Sens. + Spec.)/2      .575      .556      .594
---------------------------------------------------------------------------
Likelihood ratio (+)     Pr(+|A)/Pr(+|N)         .         .         .
Likelihood ratio (-)     Pr(-|A)/Pr(-|N)       .85      .813      .889
Odds ratio                   LR(+)/LR(-)         .        35         .
Positive predictive value        Pr(A|+)      100%     93.2%      100%
Negative predictive value        Pr(N|-)     72.1%     69.3%     74.8%
---------------------------------------------------------------------------

  Missing values or confidence intervals may be estimated
  using the -sf- or -sf0- options.

.
```

```
.
.
. **diagti   verdadeiro_positivo falso_negativo falso_positivo verdadeiro_negativo
smidf2 smaha2
.
. diagti 330 17 0 764

      True |
   disease |      Test result
    status |      Neg.       Pos. |     Total
-----------+----------------------+----------
    Normal |       764          0 |       764
  Abnormal |        17        330 |       347
-----------+----------------------+----------
     Total |       781        330 |     1,111


                                                  [95% Confidence Interval]
---------------------------------------------------------------------------
Prevalence                         Pr(A)       31%       29%      34.1%
---------------------------------------------------------------------------
Sensitivity                      Pr(+|A)     95.1%     92.3%     97.1%
Specificity                      Pr(-|N)      100%     99.5%      100%
ROC area               (Sens. + Spec.)/2      .976      .964      .987
---------------------------------------------------------------------------
Likelihood ratio (+)     Pr(+|A)/Pr(+|N)         .         .         .
Likelihood ratio (-)     Pr(-|A)/Pr(-|N)      .049     .0308     .0779
Odds ratio                   LR(+)/LR(-)         .      3753         .
Positive predictive value        Pr(A|+)      100%     98.9%      100%
Negative predictive value        Pr(N|-)     97.8%     96.5%     98.7%
---------------------------------------------------------------------------

  Missing values or confidence intervals may be estimated
  using the -sf- or -sf0- options.

.
end of do-file

. log close
      name:  <unnamed>
       log:  C:\Users\User\OneDrive\ANALISES\JULITA\Julita.log
  log type:  text
 closed on:   1 Aug 2021, 11:03:53
-----------------------------------------------------------------------------------
------------------------
```
